# Comparative Transcriptional and Genomic Analysis of *Plasmodium falciparum* Field Isolates

**DOI:** 10.1371/journal.ppat.1000644

**Published:** 2009-10-30

**Authors:** Margaret J. Mackinnon, Jinguang Li, Sachel Mok, Moses M. Kortok, Kevin Marsh, Peter R. Preiser, Zbynek Bozdech

**Affiliations:** 1 KEMRI-Wellcome Trust Research Programme, Kilifi, Kenya; 2 Department of Biological Sciences, Nanyang Technological University, Nanyang, Singapore; Weill Medical College of Cornell University, United States of America

## Abstract

Mechanisms for differential regulation of gene expression may underlie much of the phenotypic variation and adaptability of malaria parasites. Here we describe transcriptional variation among culture-adapted field isolates of *Plasmodium falciparum*, the species responsible for most malarial disease. It was found that genes coding for parasite protein export into the red cell cytosol and onto its surface, and genes coding for sexual stage proteins involved in parasite transmission are up-regulated in field isolates compared with long-term laboratory isolates. Much of this variability was associated with the loss of small or large chromosomal segments, or other forms of gene copy number variation that are prevalent in the *P. falciparum* genome (copy number variants, CNVs). Expression levels of genes inside these segments were correlated to that of genes outside and adjacent to the segment boundaries, and this association declined with distance from the CNV boundary. This observation could not be explained by copy number variation in these adjacent genes. This suggests a local-acting regulatory role for CNVs in transcription of neighboring genes and helps explain the chromosomal clustering that we observed here. Transcriptional co-regulation of physical clusters of adaptive genes may provide a way for the parasite to readily adapt to its highly heterogeneous and strongly selective environment.

## Author Summary

Malaria parasites are particularly good at adapting to the variable environments they encounter during their life cycle. This property helps explain their widespread prevalence and persistence despite massive malaria control campaigns. The genes responsible for this adaptability are largely unknown. In this study we analyzed gene expression profiles of *P. falciparum* parasites recently taken from the field and compared them to those from laboratory-adapted parasites. Many of the genes that were up-regulated in field isolates coded for proteins which are exported, or involved in export, from the parasite onto the surface of the host cell where they interact with the immune system. Differences at the expression level were partly explainable by variation in gene copy number caused by deletions or amplification of small chromosomal segments. Usually, higher gene copy was associated with elevated expression levels, but for some genes, more copies appeared to repress expression. Remarkably, the regulatory effects of deleted or amplified segments appeared to extend to genes outside the segment boundaries. This suggests an epigenetically mediated co-regulation of tightly linked gene clusters. Such ‘soft-wired’, coordinated adaptation is potentially an important mechanism by which the parasite readily adapts to its current host. The molecules responsible may therefore be important targets for drugs or vaccines.

## Introduction

Malaria is a global health problem that imposes major strain on the development of many tropical countries [1]. The challenges in combating malaria are largely due to the complexity of the parasite responsible for most of the disease, *Plasmodium falciparum*. In particular, the parasite's inherent ability to adapt to its environment, coupled with its extraordinary biological diversity can erode the efficacy of control interventions: the widespread development of drug resistance is a salient example of this [2]. Other parasite traits that are under natural selection, such as virulence (parasite-mediated damage to its host) and transmissibility (the parasite's success in spreading to new hosts), may evolve in response to control programs because they are key determinants of parasite fitness [3],[4]. Understanding the molecular mechanisms of these traits, their genetic regulation and their evolution in natural populations is fundamental to designing successful, sustained control programs and new therapies.

Whole transcriptome analyses using microarrays has opened up the possibility of simultaneously exploring all the parasite's genes for their combined and individual function, and their regulation. In *Plasmodium*, through a series of approaches such as gene knockouts [5],[6], comparative genomics and bioinformatics [7]–[12], and transcriptome-phenotype or -genotype association studies using microarrays [13]–[17], a picture is building up of the types of genes that are essential to the parasite's function and survival *in vivo* and hence important in disease processes. A further theme that has emerged from these studies is the remarkable degree of coordinated regulation in the transcription of *P. falciparum* genes, both within and between life stages [18]–[22]. This is evident from the strong co-regulation through time within the asexual cycle of genes with related function [19],[20],[22],[23], but has also been observed for genes that share similar location in the genome [9],[20],[22],[24],[25]. The mechanisms for coordinated control of transcription in *Plasmodium* are not well understood: the current focus is on chromatin modification, locus repositioning within nuclear sub-compartments, identification of DNA-binding regulatory proteins, and the role of RNA decay in regulating transcript abundance (reviewed in [26],[27]). The contribution of transcriptional regulation to whole-organism phenotypes such as virulence and transmissibility in natural parasite populations has not yet been investigated.

Several technical hurdles need to be overcome before large-scale analyses of natural parasite populations can be done. The first is to define the amount of biological and technical variability in microarray data from field parasites so that adequately powered experiments can be designed. The second is to obtain samples throughout the 48-hour cycle since most genes in *P. falciparum* have stage-specific expression [19],[20] and patient samples only yield parasites at an early stage in their cycle. This can be overcome by maturing the parasites *in vitro* and then taking samples for RNA in time series as they mature through the 48 hours. This, however, yields a third challenge in that volumes of blood from patients are usually too small to provide enough RNA for multiple time point analyses. Finally, there is some uncertainty about the adequacy of microarrays to capture the relevant variation among field parasites [28] because most arrays are designed on the basis of a single reference genome (from clone 3D7) that has been maintained in the laboratory for a long time [29].

Therefore, with a view to scaling up the application of microarray technology to studies of natural populations of *P. falciparum*, we performed whole-transcriptome analyses on a handful of field and laboratory parasites starting with the small amounts of RNA material typically obtained from a patient sample. We sampled parasite cultures at regular intervals throughout the 48-hour cycle and incorporated time point effects into the analysis. By performing technical and biological replicates we were able to quantify the level of transcriptional biological variation among field isolates relative to background technical noise , thereby testing for their true biological differences from laboratory isolates. We found that much of this variation could be explained by gene copy number variation at the sequence level due to deletion and amplification events. We further found evidence of co-regulation of genes outside as well as inside these copy number variant (CNV) regions suggesting that they act as important determinants of transcriptional regulation.

## Results

A panel of 5 recently culture-adapted field isolates of *P. falciparum*, and 3 laboratory-adapted isolates were subjected to whole-genome transcriptional analysis using a 70mer DNA microarray that contained 10,417 probes representing 5577 genes [30]. Material was taken from synchronized cultures every 8 hours for 48 hours, amplified by PCR using the SMART technique (see below), and assayed on microarrays in triplicate. The focus of our analysis was on differential expression between strains rather than differential expression through time as this has been well addressed in previous analyses of the transcriptome [19],[20],[31],[32]. To determine whether strains differed in stage of maturity at the time of harvesting RNA we used the data from the 48-hour transcriptome [19] to estimate the ‘age’ of each strain at the first sampling time point [15]: this ranged between −3hr and +4hr with a mean of 0.7hr (Figure S1). This variability in time of harvesting, despite efforts to standardize stage of maturity in our cultures, highlights the need in transcriptional studies of *Plasmodium* to take samples from multiple time points to avoid confounding of true biological differences with those due to stage of maturity [13], [14], [33]–[36].

### Evaluation of amplification method

To overcome the problem of limited amounts of parasite RNA obtainable from patients, we adapted and validated an RNA amplification technique [37] for use in *P. falciparum* that yields enough mRNA to perform multiple time point transcriptome analysis from a typical patient (<3ml of whole blood). Using strain 3D7, we tested the correspondence between expression profiles obtained using amplified vs. unamplified cDNA (Figures S2A, B). There was good correspondence between time profiles of individual genes measured using the two methods (Figure S2C) and in whole-genome expression profiles within each time-point (Figure S2B). Amplification of cDNA by SMART-PCR gave higher sensitivity to detect genes with low expression values (Figure S2), as found previously [37]. It also led to underestimation of expression in highly expressed genes: the regression slope of genes with above average expression values was b = 0.78 which is significantly (P<0.001) below the value of 1 expected if the fidelity of amplification was 100%. Thus amplification improved sensitivity but introduced some downward quantitative bias into absolute measures of expression. The magnitude of this bias was such that genes that were 8-fold lower than the average gene (i.e., the common reference material) before amplification were 4.5-fold after amplification while those that were 8-fold higher than average were 5-fold higher after amplification. However, amplification made little difference to the ranking of expression values: the Spearman rank correlation coefficient across all probes and time points in amplified vs. unamplified material was r = 0.73 which is close to that expected from within-method technical variability alone (r = 0.83 for replicate hybridizations within each method). We therefore proceeded to use this technique for analysis of RNA material from culture-adapted field isolates.

### Differences between field and laboratory strains

Of the 5224 genes represented on the array after excluding *vars*, *rifins*, *stevors*, tRNAs and rRNAs we obtained full data (all time points for all strains in at least one hybridization and one probe) on 4896 (94%) of these (Dataset S1). Across all probes and arrays, 9% of the data were excluded due to low levels of signal or poor spot quality.

**Figure 1:**
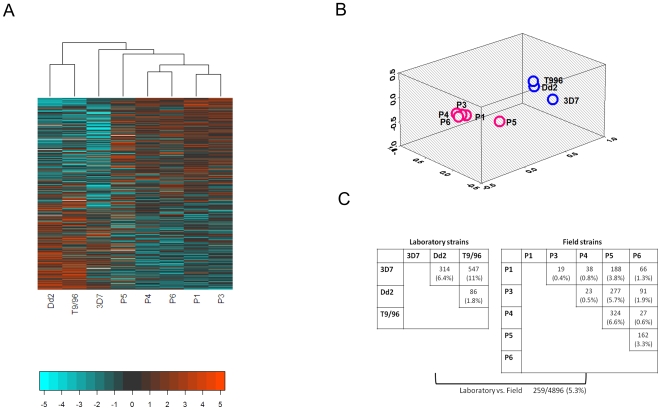
Overview of Pairwise Comparison of Transcriptional Differences between *P. falciparum* Laboratory and Field Strains. **A.** Heat map representing each strain's (column) expression values for all genes (rows). Rows are ordered top to bottom according to the maximum difference between field and laboratory isolates. Values represented by colors (red, positive; blue, negative) are summary measures over time points (averages across the life cycle, as output from the statistical model) of the strain's log_2_ expression value relative to the reference material. Values are mean-normalized across rows to highlight between-strain differences. Hierarchical clustering of the column data yielded a dendrogram that broadly separated the laboratory and field strains into two clusters with 3D7 falling somewhere between these groups. **B.** When represented by a 3-dimensional principal coordinates plot in which distances between points indicate the degree in similarity between strains across their whole genome expression profile, laboratory and field isolates fall into two distinct clusters. **C.** Tests of significance revealed that 5.3% of genes were significantly differentially expressed between laboratory and field strains (>1.5-fold difference, P<0.01 after allowing for multiple testing and including genes for which the majority of probes per gene were significant). 3D7 and P5 were more different from their counterparts than other strains.

Hierarchical clustering of the whole genome expression profiles showed clear differentiation between laboratory and field strains (Figure 1A). Principal coordinates analysis [38] confirmed these groupings (Figure 1B). After allowing for multiple testing, applying a cut-off of >1.5-fold difference based on a volcano plot (Figure S4), and taking only those genes for which the majority of their probes at the P<0.01 level, the proportion of genes that differed between laboratory and field isolates was 5.3% (259 genes, Figure 1C). For genes with multiple probes and at least one probe significant, 55% of the remaining probes were also significant: when compared with that expected by chance (6.8% of all probes were significant), this indicates high consistency in expression levels across probes representing the same gene. Of those genes declared significant, 78% were up-regulated and 22% were down-regulated in field strains compared with laboratory strains. Among field isolates alone, 592 genes (7.6%) showed significant ratios of between-strain to within-strain variability for at least one probe at the P<0.001 level: 83 (32%) of the genes that differed significantly between field and laboratory isolates also differed among field isolates.

**Figure 2:**
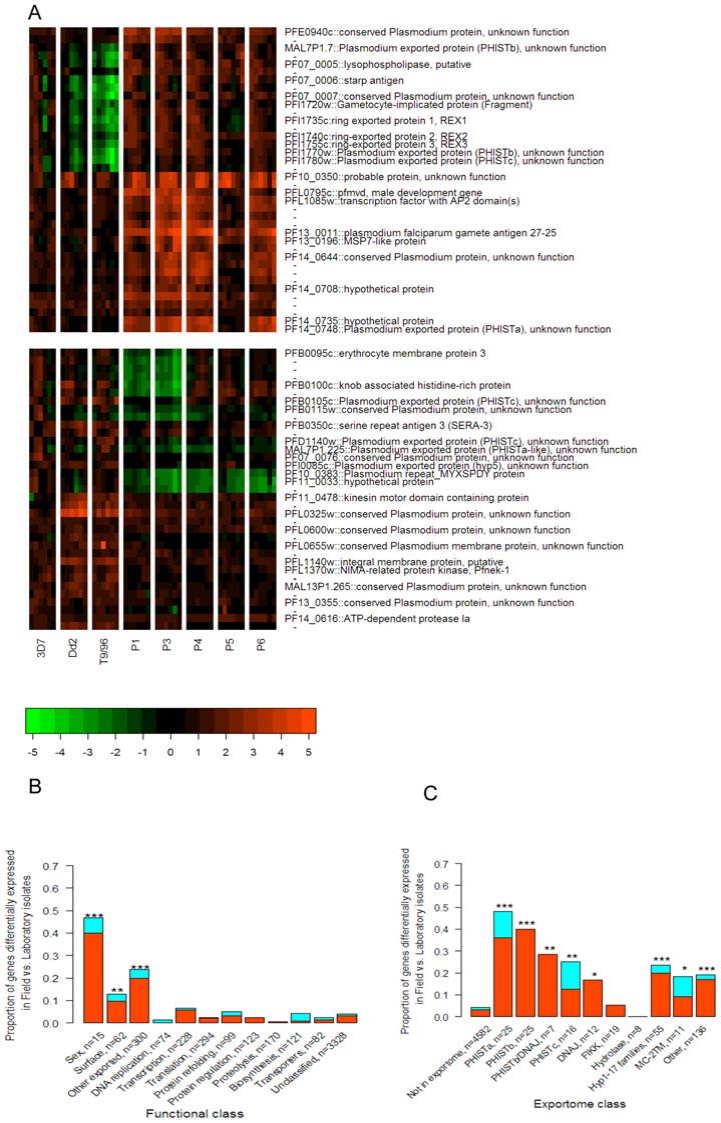
Genes that Differed Most Between Field and Laboratory Isolates and Their Functional Classification. **A.** Individual time point expression profiles for the 20 up-regulated (top panel) and 20 down-regulated genes (bottom panel) with lowest P-values in field vs. laboratory isolates comparisons. Rows are sorted by chromosomal location. All probes per gene, whether significant or not, are shown to illustrate the good correspondence across probes within gene. The data shown are actual expression values relative to the 3D7 reference material on the log_2_ scale (see color bar), i.e., they have not been row-mean normalized. **B.** Genes were classified according to function based on their PlasmoDB annotations and GO terms, and bioinformatics studies (Table S1). The proportion of each class that was differentially expressed (>1.5-fold difference and P<0.01) between field and laboratory isolates was significantly higher in genes coding for sexual stage parasites, surface proteins and genes containing an export motif (exportome) than for other genes. Differentially regulated genes tended to be more often up-regulated (red) than down-regulated (light blue). **C.** Genes were classified according to their class within the exportome [57]. With the exceptions of the Maurer's Cleft genes (MC-2TM) and the FIKK kinases, all of the sub-classes had significantly higher numbers of up-regulated in field vs. laboratory isolates than non-exportome genes (left bar). Numbers per class are shown in the axis labels. Significance levels for differences in proportions of each class vs. all other classes using the hypergeometric test are indicated by *, P<0.05; **, P<0.01; ***, P<0.001.

Figure 2A displays the time profiles of the 20 most up-regulated and 20 most down-regulated genes when comparing field vs. laboratory strains. Strain differences were highly consistent across time points and for different probes representing the same gene (Figure 2A). Notable members of the top 20 in the up-regulated group were three sexual stage-related genes (gamete antigen 27/25 (PF13_0011), a gametocyte-implicated protein (PFI1720w), and a male gamete development gene (PFL0795c)); one gene found to be suppressed in pregnancy-associated malaria (PF10_0350) [16]; a gene coding for proteins similar to the merozoite surface-expressed proteins (PF13_0196, MSP7-like protein) which sits adjacent to the MSP7 precursor that was significantly down-regulated in field isolates; a gene coding for transcription factor with AP2 DNA-binding domains (PFL1085w) which have been implicated in driving the highly coordinated regulation of transcription during the 48-hour cycle [39],[40]; three (PFI1735c, PFI1740c, PFI1755c) of four homologous genes (named *rex1*, *rex2*, *rex3*, *rex4*) found in a cluster on the end of chromosome 9 that code for ring-stage proteins that are exported into the host cell [41] and found in the vesicle-like structures called Maurer's Clefts [42] which are implicated in the transport of parasite proteins to the red cell surface [43]–[47]; three contiguous genes on the end of chromosome 7 (PF07_0005, PF07_0006, PF07_0007), one of these coding for the sporozoite surface antigen, STARP, also expressed in ring stages [48], and another coding for a lysophospholipase; and four genes that code for PHIST proteins that are members of the exportome (PF14_0748, PFI1770w, PFI1780w, MAL7P1.7). The remaining four of the top 20 were genes of unknown function on the end of chromosome 14 (PF14_0644, PF14_0708 and PF14_0735 whose neighbor (PF14_0736) was also strongly up-regulated but not part of the top 20) and on chromosome 6 (PFE0940c).

Notable members of the 20 most down-regulated genes in field isolates were five genes found previously to be down-regulated in pregnancy-associated malaria, three of which code for PHIST proteins and are members of the exportome (MAL7P1.225, PFB0105c and PFD1140w), one which is a protease (PF14_0616) and another of unknown function (PFB0115w) [15],[16],[49]; another exportome gene from the *hyp5* family (PFI0085c); another gene coding for a protein kinase (PFL1370w); two homologous genes (PF10_0383 and PF11_0033) that contain a 7-amino acid repeat unit (MYXSPDY); a serine-repeat antigen (SERA-3, PFB0350c) [5],[50]; an integral membrane protein (PFL1140w); a kinesin motor encoding gene (PF11_0478); two further genes (PFB0095c, PFB0100c) in addition to those above found on the left end of chromosome 2 which has been shown previously to be prone to a large deletion containing these genes [51]–[53], including two genes associated with knob formation [54]–[56]; and five genes of unknown function (PF07_0076, MAL8P1.210, PFL0325w, PFL0600w, PFL0655w). In addition, two mitochondrial DNA-encoded mitochondrial genes coding for cytochrome oxidase I and III were found to be up regulated in laboratory strains which indicates that the long term culture adaptation may alter the utilization of mitochondria for parasite growth (data not shown).

Thus among the genes that differed most between field and laboratory isolates, there was a preponderance of those coding for processes likely to be important for parasite fitness *in vivo* such as export of proteins to the red cell surface, merozoite surface proteins, production of sexual stage forms for transmission to new hosts, and adaptation to the pregnant vs. non-pregnant host environment. It was also noticed that many of these highly regulated genes were located in regions of the genome in which deletion and amplification events have been reported to occur. Both these aspects are explored more rigorously below.

### Functional classification of genes that differ between field and laboratory isolates

We found that genes coding for proteins involved in sexual stage parasites, those known to be expressed on the surface of the parasite, and those thought to be exported to the red cell surface membrane (members of the exportome, Table S1) were significantly over-represented among the genes significantly different between field and laboratory strains (Figure 2B). Most of these had higher expression levels in field isolates than in laboratory isolates. Other classes of genes involved in basic metabolic processes such as transporters, genes involved in replication, transcription, translation and protein fate were not well represented among genes that differed between field and laboratory isolates. When the exportome genes were classified into their sub-families [57], it was found that all of these classes were significantly over-represented in field isolates with two exceptions - those belonging to the hydrolase and FIKK protein kinase groups (Figure 2C). Since the number of genes per class was small and the overall proportion significant was low, this result cannot be strongly interpreted as lack of strain variability in expression of these genes. Nonetheless, they are notable for their contrasting pattern to other members of the exportome, and suggest that these genes are involved in biological processes essential to *in vivo* survival.

### Chromosomal clustering of differentially regulated genes

**Figure 3:**
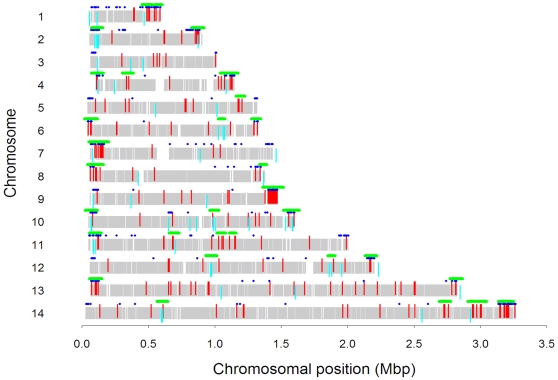
Chromosomal Location of Genes Differentially Expressed between Field and Laboratory Strains. Chromosomal locations of genes that had significantly (P<0.01, >1.5-fold difference) higher (red) or lower (light blue) expression levels in field vs. laboratory strains in at least half of their probes. Regions in which there were significantly more (P<0.05) differentially regulated genes than expected by chance (i.e., clustering) are indicated by a green bar above the chromosome. Genes belonging to the exportome are indicated by blue dots above the chromosome. Vertical grey bars indicate the locations of genes included in these analyses.

Significant chromosomal location clustering of genes that differed between field and laboratory strains was found in 30 regions of the genome; 19 of these were in the subtelomeric regions (Figure 3A, Figure S6). Clusters were also found for genes that varied significantly within field strains, 13 of 33 which overlapped with those for between-group comparisons (Figure S7). Clustering of genes that share similar timing of expression [20],[22], similar function [20],[24],[25], similar response to drugs [36] and general chromosomal location (subtelomeric vs. centromeric) [32],[36] has been reported previously. Here we show physical clustering within short chromosomal regions of genes that exhibit strain variability between strains in their expression. This implies co-regulation of genes occupying the same genomic region. Possible reasons for such clustering are through chromatin-related epigenetic control of transcription in the region [20], or the existence of deletion or amplification events in genomic DNA segments. Many reports of the latter, termed copy number variants (CNVs), have been reported previously in laboratory and fresh field isolates of *P. falciparum* based on either microarray hybridizations of genomic DNA [19],[50],[53],[58] or genetic studies of single genes or chromosomal regions [51], [52], [59]–[63]. To investigate the role of CNVs in chromosomal clustering of gene expression patterns, we mapped onto the genome all gene deletions and gene amplification events previously reported in the literature, and performed comparative genomic hybridization (CGH) of genomic DNA from our field isolates to detect further regions showing variability between strains (Dataset S2). From these data we generated a comprehensive map of genes that display copy number variation (Table S2, Figure S5). We included in this list, as a separate category, the nine ‘segmental duplications’ (SDs) consisting of four genes in tandem described by Lavazec et al. (2006) and Mok et al. (2008) which also display copy number variation [25]. A total of 448 genes in 264 separate CNVs were defined (see Materials and Methods), 154 of which have not been reported previously. CNVs displayed significant clustering in 22 regions of the genome, 10 of which overlapped with expression clusters (Figure S5).

### Associations between gene expression and gene copy number

**Figure 4:**
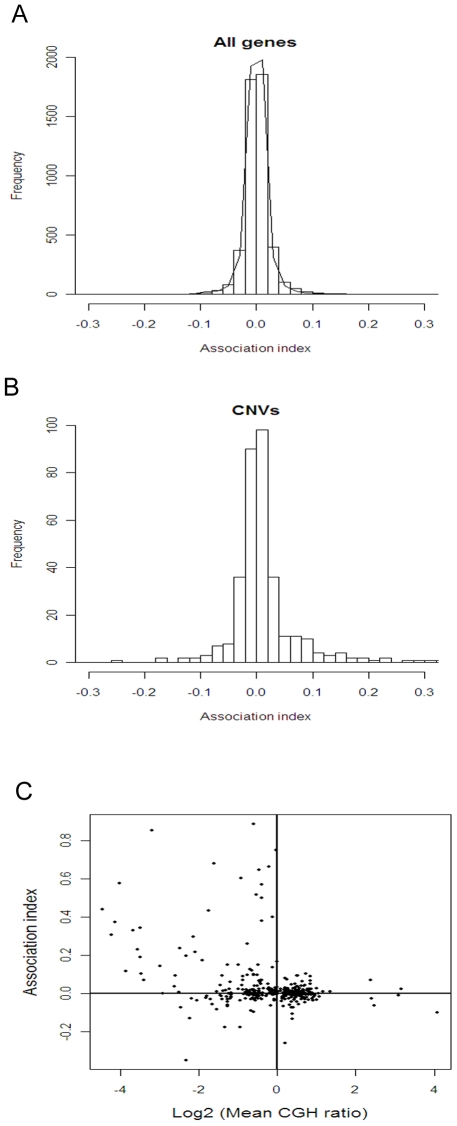
Associations Between Expression Levels and Genomic DNA Content among Field Isolates. Regression coefficients of normalized hybridization signals from cDNA on normalized hybridization signals from gDNA in the 5 field isolates were calculated for each gene. **A.** The frequency distribution of these coefficients (association index) across all genes was more dispersed than expected by chance (black line, obtained by randomly shuffling the data across isolates within each gene, P<0.001 by Kolmogorov-Smirnov test). **B.** Genes that showed variation at the genome level (CNVs) had a higher frequency of strong positive and strong negative associations than genes that did not (P<0.001 by Kolmogorov-Smirnov test). **C.** Genes with reduced genomic content relative to 3D7 had stronger associations, both positive and negative, with expression levels than genes with equivalent genomic content to 3D7. Only data from genes showing significant variation at the genomic level are shown in **C**.

We overlaid on the CNV map all the genes in our study that showed strain variation in expression levels, both between field and laboratory isolates groups and within field strains (Figure S10) and observed that many of these coincided. Therefore we jointly analyzed expression and genomic DNA (CGH) data from across field strains to determine the degree of correspondence between genomic content and expression levels. We found that across-strain associations between expression levels and genomic copy number tended to be more often strongly positive or strongly negative than expected by chance (Figure 4A, B). Positive correlations between expression and DNA copy number are generally expected because of the direct effects of gene dosage. In most cases here, positive associations appeared to be due to deletions (Figure 4C). By contrast, negative associations between expression and CGH signal for some genes indicate that lower copy number leads to higher expression. This is possibly due to their release from gene repression. We cannot say whether these apparently repressed genes had been amplified in 3D7 relative to field isolates or whether, instead, field isolates contain sequence variation in the probes for genes and therefore, from CGH data, appear to be absent. The former explanation seems more probable given that the probes for this array were designed around non-variable regions, and because only those genes for which the majority of probes showed significance, i.e., consistency across probes in the same gene, were included in the analysis. Genes showing negative associations between expression and gene copy number were more prevalent in exported genes than those not showing gene copy number variation (P<0.001 by Fisher's Exact test) but less so than for genes showing positive associations (P<0.01) These data suggest that during *in vitro* adaptation, both deletions and amplifications arise that cause, respectively, loss or suppression of gene expression in genes required for *in vivo* processes. Possible mechanisms underlying amplification-mediated repression may be negative feedback effects of the gene's protein product on its own transcription, or chromatin-mediated suppressive effects that are induced by the CNV's structure, as discussed below.

### Co-regulation between genes inside CNVs and adjacent genes

**Figure 5:**
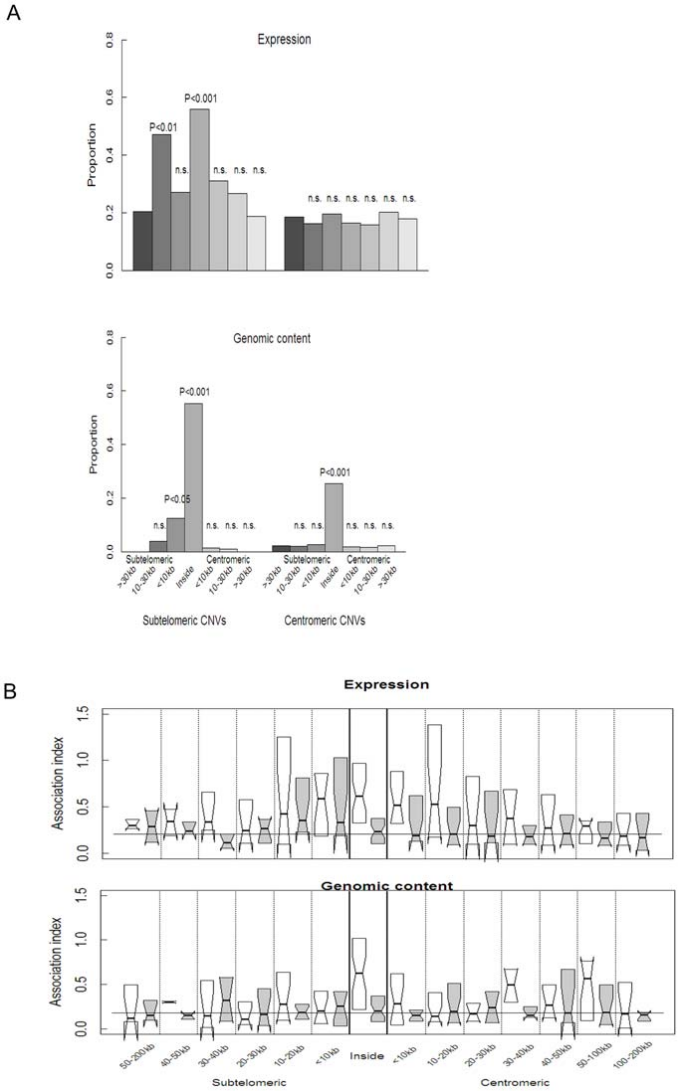
Co-regulation of Genes Surrounding CNVs. **A.** Proportion of genes significantly differentially regulated between field and laboratory isolates or among field isolates (upper), and showing significant between-strain variation at the genomic level (lower), grouped by distance of the gene from the CNV boundary on either the subtelomeric side or centromeric side. P-values (by Fisher's Exact test) indicate where the proportion of significant genes differ from that >30kb on the subtelomeric side of the CNV. Analyses have been split by whether the CNV occurs in the subtelomeric (left) or internal (right) regions of the chromosome. **B.** Across-strain (field and laboratory strains) measures of association (absolute values of regression coefficients, white bars) between expression levels (upper, white bars) of a single differentially expressed gene within a CNV (index gene) and expression levels in differentially expressed genes surrounding the CNV (test gene) grouped by distance from the CNV boundary. The same analysis was performed for genomic content data (lower). For comparison to that expected by chance, associations after randomly permuting the data across strains within genes are shown in grey boxes. The horizontal line inside each box shows the median, the box boundaries show the interquartile range, the whisker length is one interquartile range, the box width is proportional to the square root of the number of observations per group, and the notches show the approximate 95% confidence intervals, i.e., non-overlapping notches strongly support a hypothesis of non-equivalence [135]. The horizontal line across all the boxes indicates the median value for the association index of permuted values on all genes after excluding data for genes inside the CNV, i.e., the expected value of the association index from chance alone for test genes outside CNVs. Regression analysis of expression data from individual genes showed a significant decline in the magnitude of across-strain associations with distance from the CNV boundary (P<0.001) which was stronger than for randomized data (P<0.05), and was not observed for genomic content data (P>0.5). (See main text for details).

To investigate further CNV-related transcriptional regulation in relation to chromosomal clustering, we calculated the proportion of differentially expressed genes (between field and laboratory isolates and among field isolates) as a function of distance from CNV boundaries. Genes contained inside and close to CNVs located within subtelomeres, but not centromeric regions were significantly more often differentially expressed than those far from the CNV boundaries (Figure 5A). The same pattern was observed at the genomic level except that, in addition to subtelomeric CNVs, centromeric CNVs also showed (by definition) differential genomic content (Figure 5A).

We then calculated the correlations in expression levels between genes inside CNVs with those for genes outside CNVs. Associations in expression between pairs of genes both of which were located inside the same CNV were on average more positive than expected at random (Figure 5B, P<0.01 by t-test). Associations in expression levels between pairs of genes inside the CNV and outside the CNV boundary were more strongly negative or positive than by chance alone (Figure 5B), a pattern that grew weaker as distance from the CNV boundary increased (P<0.001 for the regression slope of absolute values of the observed association on distance from the CNV boundary, compared with P = 0.14 for randomized values; P<0.05 for the interaction term testing for differences between these slopes). For these tests, only differentially expressed test genes and index genes were included in the analysis: this was to rule out bias in the association index caused by the higher frequency of differentially expressed genes closer to CNVs (Figure 5A). However, when all non-differentially expressed test genes were included, these significance values were only mildly reduced. To determine whether the co-regulation around the CNVs might be the direct result of undetected gene copy number variation in regions near to the CNVs, we performed the same association-distance analysis on data from genomic DNA. While there was a strong association in gene copy number among pairs of genes contained inside the defined CNVs (P<0.001, when compared by t-test with the randomized values), no such association was found for genes outside the CNV (Figure 5B, P>0.5 for regression of associations on distance for both observed and randomized values). Thus we conclude that co-regulation of genes surrounding CNVs with those inside CNVs acts through a mechanism that is not a direct effect of genomic content of the surrounding genes.

For SDs, the pattern of co-regulation was similar to that for CNVs though more striking despite being based on many fewer observations (Figure S9B, P<0.01 for the interaction term using data from only differentially expressed test genes and all SDs whether the index gene was significant or not). This pattern was not due to the influence of adjacent CNVs (Figure 5B), of which there were a large number containing differentially expressed genes, because we excluded test genes falling insideCNVs from the analysis. Nor was the pattern due to genomic variation because the slope of association of genomic content by distance from SDs was not significantly different from zero (P = 0.5). Unlike for CNVs, however, the proportion of genes differentially regulated inside the SDs, and the strength of co-regulation among them, was relatively low (Figure S9). Nevertheless, it appears that SDs are somehow involved in co-regulation of genes that surround them.

### Large deletions

Two of the regions that showed strongest clustering and co-regulation of genes were the large deletions on the left arm of chromosome 2 and right arm of chromosome 9. Both of these deletions have been previously well documented, mainly in cultured laboratory isolates [51], [64]–[66] but also in field isolates after short-term culture [67], as in this study. Their typical breakpoints have been well mapped in earlier studies [51],[65],[68],[69]. However, in our study, three observations did not correspond to those from previous reports.

First, the region of co-regulation appeared to extend beyond the breakpoint in both deletions (Figure S8). The breakpoint in the chromosome 2 deletion is typically found in PFB0100c, the *kharp* gene: even though extensions to include its neighbor on the centromeric side, PFB0105c, have also been observed in some laboratory strains [53],[62], our expression data and CGH data on the sub-lines that also contained the deletion (Figure S8) were consistent with the breakpoint falling inside PFB0100c. However, positive correlations between expression of genes inside the deletion and outside it extended for another 32kb containing 7 genes. Similarly, in chromosome 9, our expression data concur with previous data showing that the breakpoint is in *bporf* (PFI1710w), but the positive correlations in expression in this study extended for 12kb to include 3 more genes (Figure S8).

Second, expression of genes in the far telomeric ends of both deleted regions did not seem to be abrogated (PFB0056c, PFB0070c, PFI1785w, PFI1790w, PFI1795c, PFI1800w). This is surprising because previous studies of these and other deletions have shown that chromosomal breakages in the sub-telomeres are healed by the direct joining of telomeric sequences to the breakpoint [51],[52],[60],[70] in which case all genes on the telomeric side of the breakpoint would have zero expression levels. Third, these large deletions were found in three of our five field isolates despite the essentiality of many of the genes in these regions for *in vivo* processes such as gametocyte production [67],[71],[72], cytoadherence [67],[69],[70],[73],[74], the formation of knobs [51],[54],[64],[75],[76], maintenance of cell deformability [77] and host cell remodeling for protein trafficking [41]. The chromosome 2 deletion has not previously been found in field isolates even after short-term culture adaptation [50],[52],[58],[67] although the chromosome 9 deletion has [67]. Our field isolates used for expression analyses were cultured for up to 18 weeks and for CGH data for a further 8–24 weeks (Table 1). It is probable that the mutations arose in culture and were selectively favored through having higher replication rates: the relatively high mutation rates of these deletions in culture makes this a likely possibility [78]. Alternatively, these deletions may have existed at very minor frequencies within their infected hosts in which case they would not be detectable using conventional methods immediately after isolation from the host. The fact that independent sub-lines of P1 diverged for the chromosome 2 deletion, and that the chromosome 9 deletion appeared upon further rounds of culture in all 5 field isolates (Figure S8), illustrates the particular vulnerability of parasite genomes to deletions in these regions and to their frequency changes within cultures. If the same applies to other CNVs, this highlights the need to define the biological role of CNVs in isolates taken directly from patients.

**Table 1:** *P. falciparum* Strains and Experimental Design Used in This Study.

Purpose	Strain & sub-line	Description	Method of cDNA/gDNA preparation	No. of cycles in culture	No. of replicate hybridizations
**Testing amplification method**					
	a3D7	Laboratory	Unamplified	Many	2
**Transcriptome analysis**					
	3D7	Laboratory	Amplified	Many	2
	bDd2	Laboratory	Amplified	Many	1
	cT9/96	Laboratory	Amplified	Many	1
	dP1	Field, severe	Amplified	62	3
	dP3	Field, severe	Amplified	63	3
	dP4	Field, mild	Amplified	48	3
	dP5	Field, mild	Amplified	66	2
	dP6	Field, mild	Amplified	59	3
**Gene copy number analysis**					
	dP1.A	As above	Unamplified	e+21	3
	dP3.A		Unamplified	+24	3
	dP4.A		Unamplified	+31	3
	dP5.A		Unamplified	+21	3
	dP6.A		Unamplified	+23	3
	dP1.B		Unamplified	+8	1
	dP4.B		Unamplified	+14	1
	dP6.B		Unamplified	+8	1

a Derived from a patient in the Netherlands living near the airport [131]. Believed to be of African origin.

b Obtained by cloning of strain W2-mef, a clone of Indochina III/CDC that had been under long-term mefloquine pressure [132].

c Obtained from Thailand [133],[134].

d Obtained from Kilifi, Kenya, from patients admitted to the hospital with severe or mild malaria.

e The “+” indicates the number of cycles of culture in addition to those in the top part of the table.

### Malaria-in-pregnancy genes

Of the 31 genes previously reported to be associated with pregnancy in malaria (PAM, Table S3), 9 are located within a CNV, and 19 are located outside but within 50kb of a CNV boundary. Of the 31, 13 showed differential expression between strains (11 of these between field and laboratory isolates and 2 among field strains). Those PAM genes that were up-regulated in pregnant women vs. non-pregnant hosts in other studies tended to be up-regulated genes in field isolates compared with laboratory isolates (Table S3), whereas those down-regulated in pregnant women were down-regulated in field vs. laboratory isolates. Notably, the two genes most consistently found to be up-regulated in previous studies (PFB0115w, PFI1785w) fall just outside the boundaries of the large deletions on chromosomes 2 and 9 (Figures S8, S10) and within the regions in which positive co-regulation with the CNV genes remained elevated (Figure S8). Thus these pregnancy-associated genes may be examples of *cis*-acting CNV-mediated gene regulation that allows the parasite to rapidly adapt to new, immunity-free host niches.

### Drug resistance genes

A further example of a link between CNVs and differential gene expression is the internal region of chromosome 12 where 3 of the genes inside or very close to a CNV (denoted CNV12H, Table S2) displayed significant between-strain variation in expression (Figure S10, Table S2). One of these genes (PFL1155w) codes for GTP-cyclohydrolase I (*gch1*) which is the first enzyme in the folate synthesis pathway. Higher copy number of genes in the vicinity of PFL1155w was previously reported in several laboratory strains compared with field strains by Kidgell et al. (2006) [50]. Two alternative explanations were offered for this. Either this was generated by the different composition of folate synthesis precursors in the *in vitro* culture media compared to serum, in which case the parasite would need to up-regulate its synthesis to compensate for not being able to scavenge it from the host. Alternatively, amplification in laboratory strains arose as a result of selection for antifolate resistance *in vitro*, while field isolates, in their study, had not. Recent evidence from field isolates sampled from areas with high and low antifolate drug pressure supports the second hypothesis [17]. However, our data are more consistent with the first hypothesis for several reasons. First, our isolates were obtained from an area where antifolate drugs had been in use for many years and have generated a very high level of resistance [79]. Second, we found high expression of *gch1* in 3D7, which is antifolate-sensitive, as for other laboratory isolates and consistent with the higher copy number reported by Kidgell et al. (2006) [50]. Third, examination of expression of the 8 other genes in the folate synthesis pathway and its co-pathway – the shikimate pathway - revealed that two of them also were significantly down-regulated in field isolates, viz., dihydrofolate synthase (PF13_0140, *dhfs*, P<0.05) and bifunctional dihydrofolate reductase (PFD0830w, *dhfr*, P<0.001). This suggests that there was general up-regulation of the folate synthesis pathway in laboratory isolates compared to field isolates. This support for the first hypothesis does not preclude the second also being true: direct comparisons of expression levels of this gene and copy number in resistant vs. sensitive isolates are required to determine the role of *gch1* copy number in antifolate resistance. The data do nevertheless highlight the role of gene copy number variation in parasite adaptation, of which drug resistance is just one example [17], [80]–[82], as well as the need to explore its genetic basis in natural field isolates.

## Discussion

In the process of validating microarray technology for the analysis of transcriptional variation in natural populations of malaria parasites, we found supporting evidence for many of the results from previous whole genome studies [13]–[16], [19], [20], [24], [31], [33]–[36], despite many differences in experimental design, materials and methodology. Examples of differentially expressed genes discovered in multiple independent studies include those involved in a range of biological processes important to parasite fitness *in vivo* such as export of proteins to the red cell surface, transmission, and colonization of the pregnant woman, transcriptional regulation factors, nutrient biosynthesis and metabolic pathways involved in drug resistance. This highlights the robustness of the microarray approach to the quantitative analysis of transcriptional variation in natural populations of malaria parasites.

Prior to whole genome screens, genes within several large or small deletions had been shown to be important for parasite virulence and transmissibility [51], [52], [59]–[61], [63]–[65], [67], [69], [71]–[74],[83]. With whole genome screens, many more smaller deletions and amplifications have been discovered in field and laboratory isolates [19],[31],[50],[58],[62]. Here we show that much of the biological variation in transcription between field and laboratory isolates, and among field isolates, is attributable to genes located within and around CNVs [14],[24]. We showed that gene copy number in these CNVs associates strongly with their level of expression, either strongly promoting it or suppressing it. Intriguingly, the expression levels of genes outside these CNVs are associated with expression levels of genes contained inside the CNVs. By analyzing the genomic DNA around the CNVs we were able to rule out the possibility that this co-regulation was due to undetected copy number variation in the genes outside the CNVs. We therefore suggest that co-regulation of genes adjacent to CNVs occurs through an indirect effect of the CNV.

We envisage a number of ways in which CNVs might alter the expression, or be associated with expression of genes in their vicinity. These fall under the ‘dynamic’ vs. ‘structural’ explanations for the epigenetically-mediated phenomenon of position effect variegation (PEV) that is found in many organisms (reviewed in [84],[85]). PEV refers to the maintenance and inheritance of silenced states of genes within some somatic lineages of an organism, but not others, despite there being no differences at the genetic level. Regulation of *var* genes in *P. falciparum*, in which there is mutually exclusive repression of all but one of the many genes over repeated cycles of asexual replication resembles this phenomenon [6]. The ‘dynamic’ model invokes spreading of heterochromatin from the telomeres into the adjacent regions. In *P. falciparum*, the existence of a gradated band of heterochromatin in the nuclear periphery where sub-telomeric genes are silenced [6],[86], and *cis* spreading of it into adjacent regions [87],[88] has been demonstrated. This band is considerably wider than in other organisms where this has been observed, such as yeast (∼50kb vs. ∼5kb) [6],[86], thus providing scope for simultaneous silencing of many sub-telomeric genes in *P. falciparum*. In addition to the maintenance of a heterochromatin environment, it has been shown that histone modifications alter the degree of the heterochromatin's repressive effect on genes in this region in *P. falciparum* [89]–[92]. Such heterochromatin-modifying effects, such as nucleosome assembly during replication, histone modification, DNA methylation and RNA interference are common in other organisms and are thought to maintain the reversibility of silencing by heterochromatin (reviewed in [93]). Applying the dynamic model to our results, we postulate that CNVs may act as insulators to chromatin spreading, or as modifiers to its effectiveness, as is common in other organisms (reviewed in [94]). Increased copy number in tandem repeat non-coding sequences have been shown to increase their repressive effect on neighboring genes in a diverse range of organisms, even at long range, by changing the relative position of genes and the heterochromatin regions (reviewed in [95]). It is possible that copy number variation in coding genes acts in a similar way in *P. falciparum*.

The structural model of PEV proposes that somatic pairing of homologous chromosomes (in the case of diploids) and of heterologous chromosomes causes the recruitment of heterochromatin and other DNA-binding proteins to the temporarily joined regions. If chromosomal rearrangements occur, or copy number variation in coding or non-coding regions, cause changes in the pairing alignment, this heterochromatin formation is altered, thus resulting in PEV (reviewed in [84]). Such effects can be manifest iat single loci as well as having long-range effects on multiple loci. In *P. falciparum*, formation of clusters or “bouquets” of heterologous chromosomes has been shown to occur during mitosis and meiosis [96]–[98] and to be involved in recombination amongst *var* genes, presumably in order to generate new diversity [96]. (SDs may play such a role for generating diversity in the *pfmc-2TM* genes which contain a hypervariable region that is likely to be exposed to the immune system [9]). Applying the structural model to our data, we speculate that formation of junctions between heterologous chromosomes, if they rely on multi-copy genes contained in or surrounding CNVs, may mediate the recruitment of heterochromatin to these regions and thereby modify the expression of genes in the vicinity. Sub-telomeric deletion events have been shown to disrupt bouquet formation [97]. There is also evidence suggesting the existence of transcriptionally permissive sub-compartments within the silencing zone on the nuclear periphery into which sub-telomeres might be transferred in order to reverse the silencing [6],[87],[99]. Thus a third model to explain our observations on co-regulation are that CNVs or SDs in some way interact with the boundaries of these sub-compartments, thereby altering the positioning of surrounding genes relative to the silencing/permissive zones. Much remains to be explored on the mechanisms and consequences of heterochromatin recruitment and modification in *Plasmodium*. Doing so could lead to powerful new drugs that disrupt heterochromatin-mediated regulation of important homeostatic and adaptive mechanisms in the parasite [100].

What might be the reasons for maintenance of variation in CNV genes in natural populations? The findings of this study support the view that these unstable regions in the genome are important to parasite adaptation in its *in vivo* environment and so are maintained in nature. In the case of the large deletions on chromosomes 2 and 9, it is likely that *in vitro* culture conditions relieve the parasite from the presumably costly but necessary *in vivo* exercise of exporting proteins, constructing knobs, displaying cytoadhesive molecules and variant antigens, and producing gametocytes (which cost the parasite future asexual replication). Their ready appearance under such conditions therefore suggests that they can have a growth advantage under some conditions. One of those conditions might be during the acute stage of infection of a naïve host when the short-term race against competitor parasite lineages for host cells and escape from innate immune mechanisms is of paramount importance to the parasite's fitness. Knobs are generally assumed to be essential for parasite survival because they mediate cytoadherence which, evidence in both human and other malaria species suggests, allow the parasite to avoid clearance by the spleen [101]–[107]. However, knobless parasites are able to cytoadhere to some degree [70],[108], and there may be other mechanisms of cytoadherence not mediated by knobs that help the parasite to sequester [109]. Therefore, rather than viewing knobs as being universally favored, we postulate that they have both benefits and costs which vary during the course of an infection. During the acute stage of the infection the benefit may be escape from immune attention and intrinsically higher replication rate, while at the chronic stage the benefits are likely to be the ability to express new ligands and antigenic types to keep ahead of emerging variant-specific antibodies. Thus we view the postulated maintenance of large chromosomal deletions at low frequencies within hosts as arising from a play-off between a short-term advantage to winning the within-host competition for host resources versus the long-term advantage of winning the competition to transmit between hosts. This fits with two well-established evolutionary theories of why highly virulent pathogens are seen in nature despite their fitness costs through host mortality [110]–[114], both of which have some empirical support in malaria (reviewed in [4]). Shorter CNVs, whether deletions or amplifications, may play a similar role in adaptation to niche environments as appears to be the case for some drug resistance genes ([14],[24],[50] and this study).

CNVs may also play a role in regulation of non-CNV adaptive genes that surround them. It is striking that the two genes most consistently associated with placental malaria are found on the boundaries of the chromosome 2 and 9 deletions (Figure S8) and half of those reported to be associated with placental malaria are located in other CNVs or within 50kb of them. Another example may be the deletion-mediated switching on of rosetting [78], a parasite virulence-related adhesive property [115]–[118] which, given its widespread prevalence in nature, and the fact that it varies during an infection (at least in the rodent malaria, *P. chabaudi*, [119]) and responds to immune selection [120], suggests it may play an adaptive role. There may be many such other phenotypic, adaptive consequences of alterations in gene copy number. The existence of shared molecular mechanisms associated with these deletions, such as small regulatory elements upstream of the breakpoints [51],[52],[60], similar gene inversion mechanisms to create them [60],[63], and healing of breakages by pasting of telomeric sequences that alter the positioning of the telomeres within the heterochromatin environment [97] supports the view that these mechanisms are maintained by the parasite to allow it to adapt to its variable host environments, both within an infection in the same host and across infections in hosts of different immune/pregnancy/genetic or other susceptibility states [75].

CNVs may also play a role in regulation of genes very distant to them or even on other chromosomes. A recent study of genome-wide transcription of a genetic cross between a drug resistant and drug sensitive line identified 12 hotspots in the genome containing genes that appear to regulate many other genes on different chromosomes to the CNV [24]. One of these regulators occurred in a region containing a CNV that is related to drug resistance [24]. Most of the 12 regulatory hotspots were found inside or close to CNVs. Thus it seems that CNVs might have genome-wide *trans*-acting effects. Other forms of master regulators of transcription in *P. falciparum* have been proposed to work through binding of specific DNA motifs of genes expressed at different stages of the parasite's asexual cycle, such as those in the ApiAP2 family [39],[40]. Three of this family of 26 genes fall within CNVs and four of them showed strain variability in expression in the study here. Thus combined, our data and those from previous studies present a picture of highly coordinated transcription of genes involved in functional processes related to parasite adaptation to its *in vivo* environment. Understanding the source of this co-regulation will provide clues as to how the parasite has maintained its extraordinary evolutionary success and may, perhaps, provide new targets for intervention.

## Materials and Methods

### Ethics statement

Collection of parasite samples from patients was approved by the KEMRI Ethical Review Committee.

### Parasite material and preparation of RNA and DNA

Five malaria parasite isolates (denoted P1, P3, P4, P5 and P6) were obtained from malaria-diagnosed inpatients and outpatients at the Kilifi District Hospital, Kenya, and adapted to *in vitro* conditions through continuous culture for 48–66 cycles (Table 1). During culture adaptation and for the experiments reported here, parasites were grown at 2–3% hematocrit with 0.5% Albumax II as a replacement for human serum and 0.1mM hypoxanthine in the standard malaria culture media [121]. At the point of collection from the patient the minimum number of genotypes per isolate were 3, 1, 3, 2 and 1 for P1, P3, P4, P5 and P6 respectively, as determined by genotyping at the MSP-2 locus [122] but appeared to consist of a single genotype by the time the experiments here were performed. Cultures of these lines were expanded to a volume of 3–4ml packed cell volume (PCV), synchronized by sorbitol treatment in two consecutive cycles, and early in the following cycle were diluted to 1% parasitemia with fresh red blood cells and split into six separate flasks containing 75ml and 0.25ml PCV. At six 8-hour intervals starting at approximately 4 hours post-invasion, cells were washed once in warm PBS, snap-frozen on dry ice, then stored at −80°C. Similar preparations were made from the laboratory strains 3D7, Dd2 and T9/96.

RNA was prepared from frozen samples after lysing them in a water bath at 62°C for 3 minutes, and then adding 2.5ml of Trizol (Invitrogen) while keeping on ice. After 10 minutes of mixing by pipetting, the RNA was extracted using two chloroform treatments, followed by overnight precipitation with isopropanol/sodium acetate precipitation. An average of 16µg (range 2–64) of RNA was obtained from each 0.25ml PCV culture sample and used at an average concentration of 0.8µg/µl in the subsequent amplification step. A pool of material from 3D7 for use as the common reference in the microarray hybridizations was constructed by pooling equal quantities of RNA collected at the 6 time-points.

Genomic DNA for comparative genomic hybridization (CGH) of the field isolates to the array was obtained by growing up each line used in the transcriptome analyses for a further for 8–24 culture cycles (Table 1). Two cultures of each line were maintained independently by two different people to create “sub-lines”. Competitive hybridizations against clone 3D7 of one set of independent set sub-lines from all 5 strains, and 3 strains from the other set, were performed using standard methods [19] in triplicate for the first set of sub-lines and in one replicate for the second set. Strain identity was confirmed using the hybridization patterns of the hypervariable *rifin*, *var* and *stevor* genes.

### Amplification of RNA using template switching and microarray hybridizations

For analyses of the transcriptome, we modified the previously reported method Switch Mechanism at the 5′ end of Reverse Transcription (SMART) [37] to obtain sufficient target cDNA for microarray assays from as little as ∼100 ng of total RNA. Briefly 100ng of total RNA was subjected to reverse transcription that was primed by two oligonucleotides (pdN9-tag, 5′-AAG CAG TGG TAT CAA CGC AGA GTN NNN NNN NN-3′ and dT25-tag, 3′-AAG CAG TGG TAT CAA CGC AGA GTA C (T)_25_ -3′) using Powerscript (Clontech) for reverse transcription. To compensate for the AT rich bias of the *Plasmodium* genes, the dNTPs mix was enriched for A-T at a ratio of 2∶1. After the first-strand synthesis, the generated cDNA was used in a second PCR with the tag oligonucleotide (3′-AAG CAG TGG TAT CAA CGC AGA GTA C-5′) and a hybrid DNA∶RNA (d(AAG CAG TGG TAT CAA CGC AGA GTA CGC)r( GGG )). In this step, aminoallyl dUTPs were incorporated into the dNTP mix (dATP∶dTTP∶aadUTP∶dCTP∶dGTP = 2∶1∶1∶1∶1∶1) for the subsequent coupling of fluorescent dies. cDNA was hybridized to a 70mer oligonucleotide microarray [30] as previously described [19]. Hybridizations were performed in triplicate or duplicate for all isolates except Dd2 and T9/96 for which single replicates were performed. Microarrays were scanned with a GenePix 4000B scanner and the images analyzed using GenePix Pro 3.0 software (Axon Instruments, Union City, CA).

### Pre-analysis of expression and CGH data

The initial raw microarray data were first filtered by eliminating poor quality spots (manual inspection) and low signal spots (median signal in both Cy3 and Cy5 channel > median background signal +3 standard deviations of the background signal as defined by the GenePix software. For expression data, in order to standardize the variance and mean in expression ratios across the range of background intensities, the “normexp” [123],[124] and the “robustspline” methods from the Limma package [124] were applied to each array separately (within-array normalization) (Figure S3). The data were then variance-normalized between arrays using the “scale” method in Limma because it was found that the variability for 3D7 was much lower than for other strains [125] (Figure S3). The reason for this lower variance was because of the positive correlation, and hence restricted variance, between the expression ratios of the sample and pooled reference material as the latter was created by pooling the former. For CGH data, it was found necessary to normalize differently to that used for expression data because of the zero hybridization levels for some genes compared with 3D7. Within-array normalization was performed by subtraction of background intensities from foreground intensities and between-array normalization was performed using the “quantile” method to ensure equal empirical distributions across arrays.

Data from the hypervariable gene families (*rifin*, *var* and *stevor*, and fragments and pseudogenes of these, collectively termed variant surface antigens, VSA) were excluded from the analysis prior to normalization because many were not recognized by the probes which were based on 3D7 VSA sequences. Data from genes coding for ribosomal RNAs and transfer RNAs were also excluded because of potential error caused by differences in the dynamic range of the signal.

All statistical analyses and graphs were generated using the R programming language [126]. The raw filtered data have been deposited in the Array Express database (www.ebi.ac.uk/microarray-as/ae/).

#### Validating the RNA amplification method

To describe the faithfulness of SMART amplification in reproducing expression differences, we compared expression profiles of all genes using cDNA prepared directly from large cultures by the usual single-step first-strand synthesis method (“Unamplified”) to that from amplified material using SMART. To examine the effects of amplification on time profiles of expression of the same gene, Pearson correlation coefficients between the two methods were calculated across the six time-points for each probe. To examine the effects of amplification on gene expression levels relative to all the other genes, Pearson correlations and Spearman rank correlations between the two methods (Unamplified vs. Amplified) were calculated across all probes within each of the 6 time points. The observed across-time point and across-gene correlations were compared to distributions of correlations obtained after 1000 random permutations of the data across time points and genes respectively. Regression analysis of data from amplified on unamplified material from all probes and time points was used to determine whether quantitative differences between genes were faithfully reproduced by amplification. For these analyses, data on the *rifins*, *vars* and *stevors* were retained as we wanted to determine whether there was amplification bias in genes with very low relative expression levels.

#### Estimating the time zero for each strain

Since each strain was synchronized and harvested separately there were potential differences in the stage of maturity (phase) of each strain at the starting time point. Therefore we estimated the phase of each strain by finding the minimum mean Euclidean distance between data on 6 time points in our strains and data from the 48-hour transcriptome data set [15],[19].

#### Testing for differences in expression among strains

To statistically test for differences among strains, a linear model with fixed effects for time point and strain was fitted to data on each probe separately using the Limma package in R [124]. A block effect was included in the model to allow for correlations between data from repeat hybridizations of the same cDNA material for each strain-time point combination [127]. For each probe, contrasts were formed between each pair of strains, and between the grouped laboratory vs. grouped field strains. These differences were tested for statistically significant deviations from zero using modified t-tests and F-tests in which, instead of using the error variance for each probe, the pooled error variance across all probes, estimated using a Bayesian approach [128], is substituted. P-values on these modified t-tests were adjusted for multiple testing using the Benjamini and Hochberg [129] (BH) method which gives the required false discovery rate, i.e., the rate of false positives among the declared positives. For the comparison of field vs. laboratory isolates, those probes with P<0.01 and a fold-change of greater than 1.5 (i.e., 50% up- or down-regulation) were declared significant. For declaring significant variation among field isolates, a cut-off value of P<0.001 by F-test was used. For secondary analyses that required data on a gene-wise rather than probe-wise basis, only genes with greater than 50% of their probes were taken to be significant.

The strain coefficient from the linear model above was used as a summary measure for each strain: this coefficient represents the average expression level across time-points. Hierarchical clustering of these summary data was applied to whole-genome expression profiles to obtain a strain dendrogram that represented relative differences among strains. Differences among strains were also described using a principal coordinates plot [38] which represents dissimilarities among strains as distances in a strain ‘map’. To create this map, the matrix of Pearson correlations between each strain's whole-genome profile were computed, and the Euclidean distances from this matrix were used as the similarity measure in a multidimensional scaling analysis using the “cmdmds” command in R [126]. Here we used a 3-dimensional map because it explained considerably more of the variation than a 2-dimensional map.

#### Testing for differences in genomic content among strains by CGH and definitions of copy number variants (CNVs)

As for expression data, a model was fitted to each gene with a fixed effect for each sub-line (8 lines representing 5 isolates) with allowance for correlations among replicate hybridizations. Genes with F-tests significant at the P<0.001 level after correcting for multiple comparisons and at least two lines different from 3D7 by at least 1.5-fold in the majority of probes per gene were declared as a copy number variant (CNV). Significant genes that were adjacent to other significant genes in this study or previously published CNVs were assigned to the same CNV (Table S2).

#### Testing for chromosomal clustering of differentially expressed genes

To examine for chromosomal clustering of genes that were differentially expressed between field and laboratory strains, for each chromosome a sliding window average expression difference was calculated using a window width of 50kb and an increment of 5kb. To statistically test for clustering, the probability of getting equal or greater than the observed number of significant genes within the window, given the number of genes that fell within that window and the proportion of all genes in the genome that showed significance (excluding those in this window), was calculated from the hypergeometric distribution, i.e. sampling from a finite pool of differently labeled objects without replacement. Clustering was declared to be present if this probability fell below P<0.01. These clustering tests were performed on both field vs. laboratory isolate comparisons and comparisons among field isolates.

#### Testing for associations between differential expression and gene function

Genes were classified according to functional classes using the gene ontology (GO) annotations obtained from version 5.5 of PlasmoDB. The genes were further classified based on lists compiled from specialist studies on genes with related functions, viz., the proteins involved in membrane transport (the “permeome” [11]), protein kinases (the “kinome” [12]), and proteins exported out of the parasite into the host cell either into the parasitophorous vacuole, the erythrocyte cytosol or the red cell surface (the “exportome”, [7],[8],[57],[130]). The criteria used for classification are given in Table S1. When genes fell into more than one class, they were assigned to the class at the highest level in Table S1. Over-representation of significantly differentially expressed genes in these classes relative to all other classes was assessed using the hypergeometric test.

#### Analysis of co-regulation

To determine whether there were associations between the expression of genes inside CNVs and SDs with other genes inside them and genes outside their boundaries, the across-strain (field and laboratory isolates) correlations and regression coefficients were computed for pairs of data from an index gene inside the CNV or SD (chosen to be that with the lowest P-value for the test of a difference between field and laboratory isolates in the case of the CNV, and the *hyp4* gene in the case of the SDs) and all other genes 200kb either side of the CNV boundary (test genes). The absolute value of the regression coefficient was taken as a measure of the strength of association and was analyzed by linear regression as a function of distance from the CNV or SD boundary. The same analysis was performed on CGH data (using data from all eight sub-lines of the field isolates) to determine whether any associations seen in expression data were attributable to variation at the genomic level.

## Supporting Information

Figure S1 Estimation of the Timing of Each Strain Relative to the 48-hour Transcriptome. For each strain and each gene, Euclidean distances between the 6-timepoint expression profiles from this study and the quality-controlled data set from the 48-hour transcriptome [19] were calculated for each of the 48 hour time points (x-axis). The mean Euclidean distance across all genes is on the y-axis. Where this distance reaches a minimum gives the best fit of the data to the reference set, i.e., the best estimate for the stage of maturity at which the samples were harvested at the first time point. Due to variation in the reference data set, there were systematic differences (i.e., across all strains) between time points in the distance measure. Therefore the curves were smoothed using a quadratic loess fitting method with an effective number of parameters of 10. The minima were then found from the smoothed curves. The best estimates ranged from 45h (−3h) to 4h with an average of 0.67h across strains. Therefore the first time point is labeled as zero hours in all other figures showing time profiles of expression data.
(2.17 MB TIF)

Figure S2 Evaluation of the SMART RNA Amplification Technique for Use in Whole-Transcriptome Analyses of *P. falciparum*. Genome-wide gene expression levels were analyzed by DNA microarrays using parasite material taken from a synchronized culture of the laboratory-adapted strain, 3D7, every 8 hrs during the parasite's 48hr asexual cycle. cDNA for hybridizing to the array was prepared from total RNA either using the normal first strand reverse transcription method (‘Unamplified’) or by SMART-PCR (‘Amplified’) (see Materials and Methods). A. Heat maps of gene expression levels across time points (x-axis) using Unamplified and Amplified material demonstrates faithful reproduction of gene expression time profiles after amplification. Only probes exhibiting at least 2-fold change during the 48hr cycle are included in the heat map. Rows (probes) are ordered according to expression levels at the first time point (0hrs) in the Unamplified material. Expression values are mean-normalized across rows. Relative expression values on the log_2_ scale are shown in the color bar below the plots. B. Scatterplot of expression values over all probes and time points using Unamplified (x-axis) and Amplified (y-axis) material. Density shading has been used to replace individual points in high density regions: 5000 individual points are shown in the least dense areas. Slopes of regression lines (dashed lines) fitted separately to data with x-values above and below zero were both significantly (P<0.001) lower than the expected value of 1 (solid line) indicating that RNA amplification leads to underestimation of relative expression differences. The boxed area indicates the few genes that were markedly preferentially amplified. Closer analysis revealed that these contain repetitive sequences that are also shared across genes: therefore their data were excluded from the main analyses. C. The frequency distribution of observed Pearson correlations between each probe's expression time-profile using Unamplified vs. Amplified material (bars) shows high correspondence between time profiles for the two methods. 57% of the probes had correlations greater than 0.8. Permutation analysis (solid line) found that this would occur by chance 3.6% of the time. D. Distributions of observed correlations between Unamplified and Amplified material across all probes at each time point ranged from 0.76 to 0.92 (solid dots for Pearson correlations, open circles for Spearman rank correlations, increasing in y-value from first to last time point). These were much higher than the range of −0.03 to 0.03 expected by chance (solid line, Pearson correlations obtained by permutation analysis). *Note for Figure S2*. A small subset of probes appeared to be preferentially amplified in strain 3D7 (Figure 1B). Of the 17 data points highlighted as over amplified, 15 of these fell within 2 time points, and they represented 11 genes. Since these over-amplification events were observed in both hybridizations, and these events were restricted to 2 time points and were consistent across multiple probes per gene, it seems likely that they arose as an artifact of the PCR conditions during amplification. Of the 11 genes showing amplification, four were paralogous genes coding for hypothetical proteins (MAL8P1.335, PFC1125w, PFC0002c, and PFD1250w). The two probes for these genes all had very similar sequences, and so it is likely that these genes were cross-hybridizing. These genes also contain repetitive sequences that are similar which may also have contributed to over-amplification. As these genes do not appear in the sequence database of strains other than 3D7, and were not expressed in strains other than 3D7 (our results), it is concluded that these were not real genes but are instead artifacts in the 3D7 genome annotation. They were therefore ignored in further analyses. Another gene that was over amplified, PF10_0374 that codes for a large gametocyte-expressed protein Pf11-1 and has 10 probes, had multiple probes that displayed over amplification. This gene contains a repetitive sequence that evolves rapidly to produce strain variation [136]–[138] and which is also found in other antigens (antigen 332 PF11_0507 and RESA). Thus the expression differences based on this gene should be considered unreliable, as supported by discrepant results for this gene in 3D7 from other studies [50],[58]. Probes for the remaining 6 genes displaying over-amplification (MAL8P1.38, PFL1210w, PF11_0330, PF11_0462, PF14_0107, and PF14_0235) showed no homology with other sequences in the database and so cannot be explained. However, only one of these (PF11_0462) showed significant differences between strains (higher in field than laboratory isolates, even when 3D7 was ignored) and so these genes were retained for further analysis. Nevertheless, the existence of amplification bias in a few genes highlights the need for highly specific oligonucleotide probes that avoid repetitive or low complexity regions [19], and for independent verification of genes that show expression differences using unamplified material [58].
(3.53 MB TIF)

Figure S3 Quality Control and Normalizations. A. Expression ratio (y-axis) vs. mean intensity (x-axis) plots from 1 array on one field isolate before and after the “normexp” normalization and “robustspline” within-array normalization procedures were applied [123],[124]. Data points shown in black are those that were filtered out due to poor quality spots or screened out because they coded for VSAs or non-messenger RNA species. Data points shown in red are those probes included in the final analysis. B. Box-plots of data from each of 108 arrays before and after between-array normalization using the “scale” method in Limma [124]. Arrays 41 and 70 were excluded from analyses due to poor quality hybridizations. Slides 85–96 are for strain 3D7 (6 time-points, 2 replicates each).
(5.38 MB TIF)

Figure S4 Volcano Plot Showing Relationship Between Expression Differences and P-values in Comparison of Field vs. Laboratory Isolates. Individual points are for probes and there are multiple probes per gene. The significance criteria of 1.5-fold expression and P<0.01 are indicated by the vertical and horizontal solid dashed lines, respectively, not allowing for multiple testing. After allowing for multiple testing, 6.8% (647/9493) of probes were significant. After including only genes for which the majority of their probes were significant, 259/4896 (5.3%) of genes were declared significant.
(0.57 MB TIF)

Figure S5 Chromosomal Location of CNVs and SDs and Functional Classification of Genes Contained Within Them. A. Chromosomal locations of genes that had significant variation among field isolates (P<0.01, >1.5-fold difference in at least two field isolates and the majority of their probes significant by these criteria) in genomic DNA content as measured by CGH. Pink bars indicate those CNVs detected in this study and brown bars indicate CNVs reported in previous studies (Table S2). Orange bars indicate the locations of SDs. Regions in which there were significantly more (P<0.05) CNVs than expected by chance (i.e., clustering) are indicated by a dark green bar above the chromosome. Clusters found in expression data (Figure 3) are shown in light green for comparison. Vertical grey bars indicate the locations of genes analyzed in this study. B. Genes were classified according to function based on their PlasmoDB annotations and GO terms, and bioinformatics studies (Table S1). The proportion of each class that were located in CNVs are shown for new CNVs found in this study (pink) and CNVs reported from previous studies (brown). C. As for B but classified according to their class within the exportome [57]. Numbers per class are shown in the x-axis labels. Significance levels for differences in proportions of each class vs. all other classes using the hypergeometric test are indicated by *, P<0.05; **, P<0.01; ***, P<0.001.
(1.90 MB TIF)

Figure S6 Sliding Window Plots of Average Expression Differences (log_2_ scale) Along Each Chromosome. Differences in expression between field vs. laboratory strains averaged over a sliding window of a 0.5Mbp region are shown as solid lines. The horizontal line marks the expected value under the null hypothesis of there being no difference between the groups. Expression differences for individual genes that were significant (P<0.01, >1.5-fold change) are shown as dots. Regions where there were significantly more genes than expected by chance (P<0.01) after allowing for gene densities are indicated by horizontal green bars. The vertical bars in the top of each panel represent the locations of all the genes in the genome.
(1.90 MB TIF)

Figure S7 Chromosomal Location of Genes Differentially Expressed within Field Strains. Chromosomal locations of genes that showed significant variation (P<0.001 by F-test after correcting for multiple comparisons and which had the majority of their probes significant) in expression levels among field strains (black bars). Regions in which there were significantly more (P<0.05) differentially regulated genes than expected by chance (i.e., clustering) are indicated by a yellow-green bar above the chromosome. Genes belonging to the exportome are indicated by blue dots above the chromosome. Vertical grey bars indicate the locations of genes included in these analyses.
(1.70 MB TIF)

Figure S8 Gene Expression Levels and Genomic DNA Content for Each Parasite Isolate for Genes in the Region of the Chromosome 2 and 9 Deletions. Expression levels (left axis, upper panel) are expressed as differences from the mean over all time points for strain 3D7. Genomic content levels (lower panel) are expressed relative to 3D7 and are shown only for field isolates. The vertical dotted line indicates the site of the breakpoint as reported in previous studies. For Chromosome 2, based on expression levels, the deletion appears to have occurred in strains P1 and P3 beginning at PFB0100c (*kharp*) or PFB0105c as reported previously (marked by a vertical dashed line) and extends through to PFB0075c. This was confirmed by CGH in sub-lines P1.A and P3.A. In Chromosome 9, from the expression data, the laboratory strains Dd2 and T9/96 appear to have a deletion between genes PFI1710w (*bporf*) and PF1780w, consistent with previous reports of the breakpoint [41]. Strain P5 appears to be carrying a mixture of deleted and non-deleted parasites. These breakpoints were confirmed in CGH analysis of the field strains, all of which carried the deletion in at least one sub-line after further culturing. In the expression data (upper panel), the black dashed line shows the across-strain correlations (right axis) between expression profiles for an index gene inside the deleted region (PFB0105c or PFI1780w) and other genes in the region. The correlations remain positive beyond the deletion boundaries, indicating co-regulation of genes neighboring the deletion. Two strongly down-regulated genes adjacent to the deletion boundaries, PFB0115w and PFI1785w, have been shown in previous studies to be up-regulated in parasites obtained from pregnant women (Table S3).
(2.45 MB TIF)

Figure S9 Co-regulation of Genes Surrounding SDs. A. Proportion of genes significantly differentially regulated between field and laboratory isolates or among field isolates (upper) and showing significant between-strain variation at the genomic level (lower) grouped by distance of the gene from the SD boundary on either its subtelomeric side or centromeric side. P-values (by Fisher's Exact test) indicate where the proportion of significant genes was different from that >30kb centromeric to the SD boundary (right-hand bar in each group of bars). Results are shown with (left) and without (right) data from genes contained within CNVs that are not also SDs. B. Across-strain (field and laboratory strains) measures of association (regression coefficients, white bars) between expression levels (upper) of a single gene within a SD (the index gene, *hyp4*, or for SD11LA, gene PF11_0015, not necessarily differentially expressed) and expression levels of genes surrounding the SD (test gene, not necessarily differentially expressed) grouped by distance from the SD boundary. The same analysis was performed for genomic content data (lower). To compare these results with those expected by chance, associations after randomly permuting the data across strains within genes were computed (grey bars). The horizontal line inside each box shows the median, the box boundaries show the interquartile range, the whisker length is one interquartile range, the box width is proportional to the square root of the number of observations per group, and the notches show the approximate 95% confidence intervals, i.e., non-overlapping notches strongly support a hypothesis of non-equivalence [135]. Regression analysis of the data from individual genes, using only gene that were differentially expressed in order to rule out bias due to higher numbers of significant genes nearer to SDs, showed a significant decline in the strength of association with distance from the SD boundary (P<0.001). This decline was stronger than for data that had been randomized (P<0.01) for expression data. A decline in association strength with distance from the SD in genomic content data was not observed (P>0.5). (See main text for details).
(4.01 MB TIF)

Figure S10 Regional Genomic Maps of CNVs, SDs, Differentially Expressed Genes, Expression Clusters, CNV Clusters and Pregnancy-Associated Genes from This and Other Studies. Grey bars indicate the locations of those genes analysed in this study. Superimposed black bars mark those genes differentially regulated within field isolates. In the next row up, red and light blue bars indicate genes up- and down-regulated genes in field vs. laboratory isolates, respectively. Pink bars in the third row mark CNVs found in this or other studies. In the fourth row orange bars represent SDs. Steel blue bars in the fifth row up show genes reported to be associated with placental malaria. Blue dots below the first row indicate exportome genes. Green horizontal lines marks regions where significant clustering (P<0.05) of genes differentially expressed between field and laboratory isolates were found, while yellow-green lines indicate significant clustering of CNVs.
(0.14 MB PDF)

Table S1 Terms used for classifying genes according to function. String searches were applied to the fields of Product Description, GO Annotated Function, GO Annotated Process and GO Annotated Component downloaded from the list of updated annotations in PlasmoDB version 5.5.
(0.05 MB DOC)

Table S2 CNVs and SDs identified in this and other studies.
(0.82 MB PDF)

Table S3 Differential expression results from this study for genes associated with pregnancy malaria in previous studies.
(0.07 MB DOC)

Dataset S1 Raw data from microarray hybridization of cDNA from 3 laboratory and 5 field isolates from *P. falciparum* taken at 6 time points during the intra-erythrocytic cycle.
(4.59 MB ZIP)

Dataset S2 Raw data on microarray hybridizatons of genomic DNA for 5 field isolates of *P. falciparum*.
(0.98 MB ZIP)
