# Supplementary figures and images for: Comparative Transcriptional and Genomic Analysis of Plasmodium falciparum Field Isolates
Source: PLoS Pathog. 2009 Oct 30;5(10):e1000644. doi: 10.1371/journal.ppat.1000644 (PMC2764095; doi:10.1371/journal.ppat.1000644)

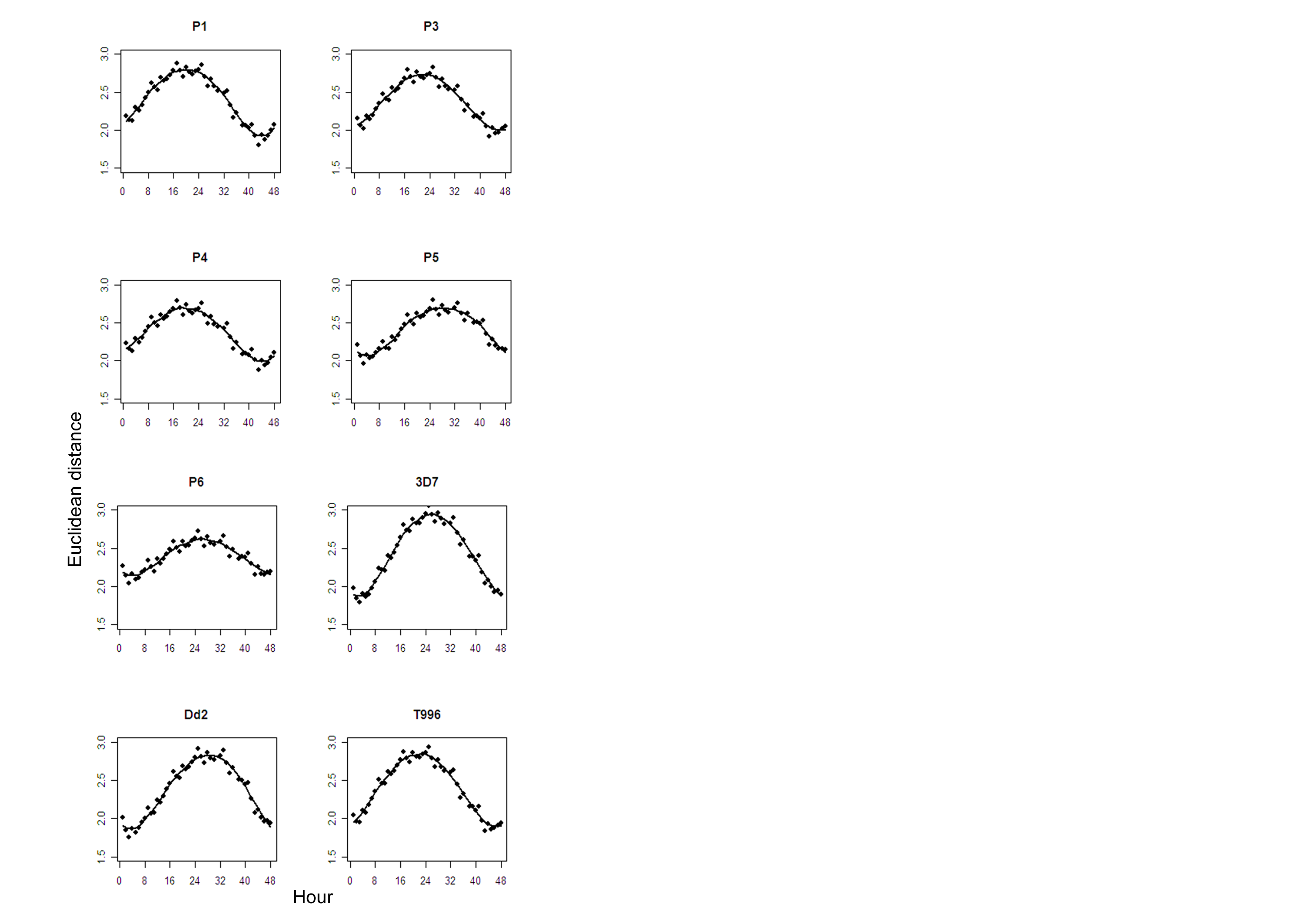

Supplement: Figure S1 — Estimation of the Timing of Each Strain Relative to the 48-hour Transcriptome. For each strain and each gene, Euclidean distances between the 6-timepoint expression profiles from this study and the quality-controlled data set from the 48-hour transcriptome [19] were calculated for each of the 48 hour time points (x-axis). The mean Euclidean distance across all genes is on the y-axis. Where this distance reaches a minimum gives the best fit of the data to the reference set, i.e., the best estimate for the stage of maturity at which the samples were harvested at the first time point. Due to variation in the reference data set, there were systematic differences (i.e., across all strains) between time points in the distance measure. Therefore the curves were smoothed using a quadratic loess fitting method with an effective number of parameters of 10. The minima were then found from the smoothed curves. The best estimates ranged from 45h (−3h) to 4h with an average of 0.67h across strains. Therefore the first time point is labeled as zero hours in all other figures showing time profiles of expression data. (2.17 MB TIF) [file ppat.1000644.s001.tif]

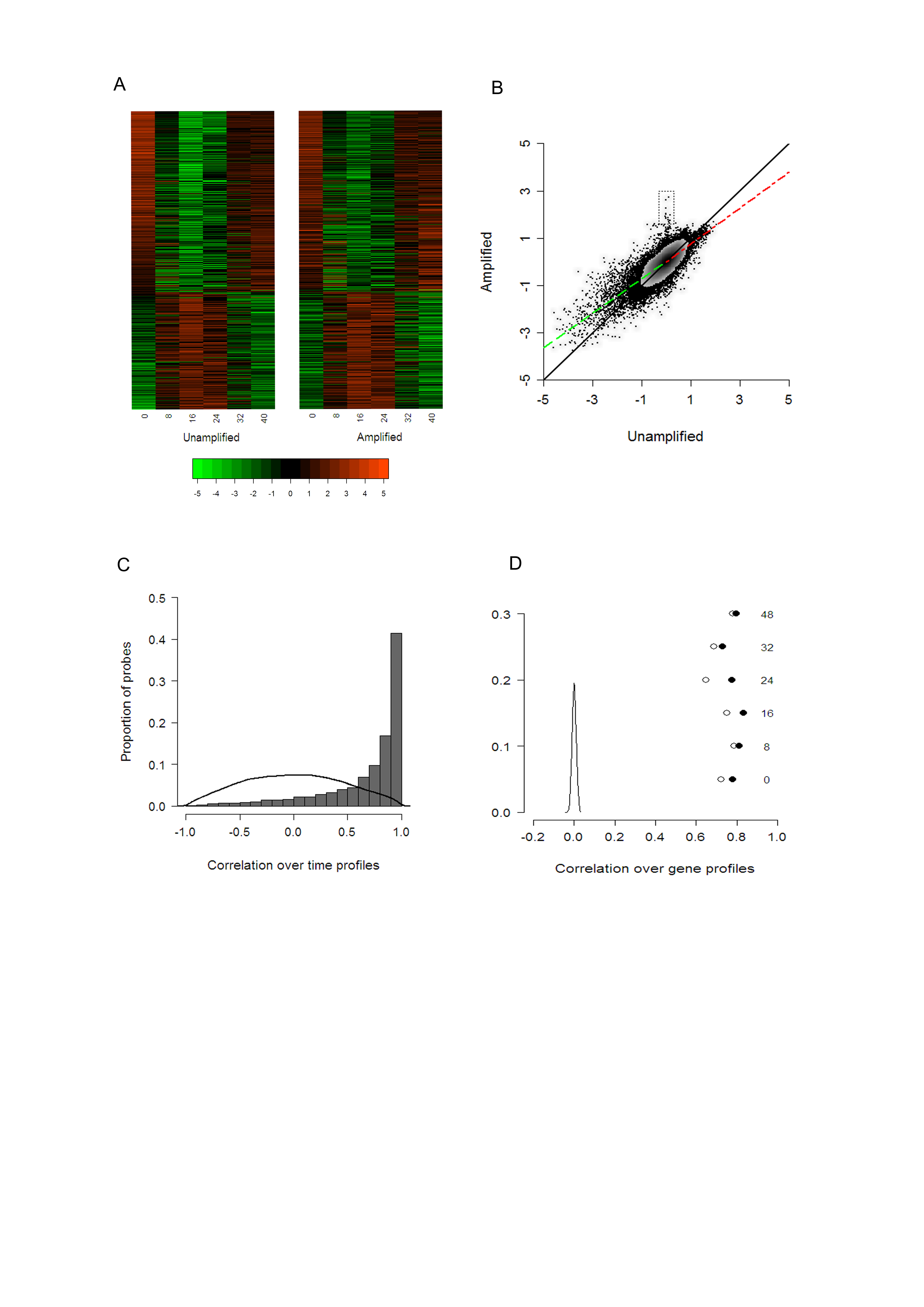

Supplement: Figure S2 — Evaluation of the SMART RNA Amplification Technique for Use in Whole-Transcriptome Analyses of P. falciparum. Genome-wide gene expression levels were analyzed by DNA microarrays using parasite material taken from a synchronized culture of the laboratory-adapted strain, 3D7, every 8 hrs during the parasite's 48hr asexual cycle. cDNA for hybridizing to the array was prepared from total RNA either using the normal first strand reverse transcription method (‘Unamplified’) or by SMART-PCR (‘Amplified’) (see Materials and Methods). A. Heat maps of gene expression levels across time points (x-axis) using Unamplified and Amplified material demonstrates faithful reproduction of gene expression time profiles after amplification. Only probes exhibiting at least 2-fold change during the 48hr cycle are included in the heat map. Rows (probes) are ordered according to expression levels at the first time point (0hrs) in the Unamplified material. Expression values are mean-normalized across rows. Relative expression values on the log2 scale are shown in the color bar below the plots. B. Scatterplot of expression values over all probes and time points using Unamplified (x-axis) and Amplified (y-axis) material. Density shading has been used to replace individual points in high density regions: 5000 individual points are shown in the least dense areas. Slopes of regression lines (dashed lines) fitted separately to data with x-values above and below zero were both significantly (P<0.001) lower than the expected value of 1 (solid line) indicating that RNA amplification leads to underestimation of relative expression differences. The boxed area indicates the few genes that were markedly preferentially amplified. Closer analysis revealed that these contain repetitive sequences that are also shared across genes: therefore their data were excluded from the main analyses. C. The frequency distribution of observed Pearson correlations between each probe's expression time-profile using Unamplif [file ppat.1000644.s002.tif]

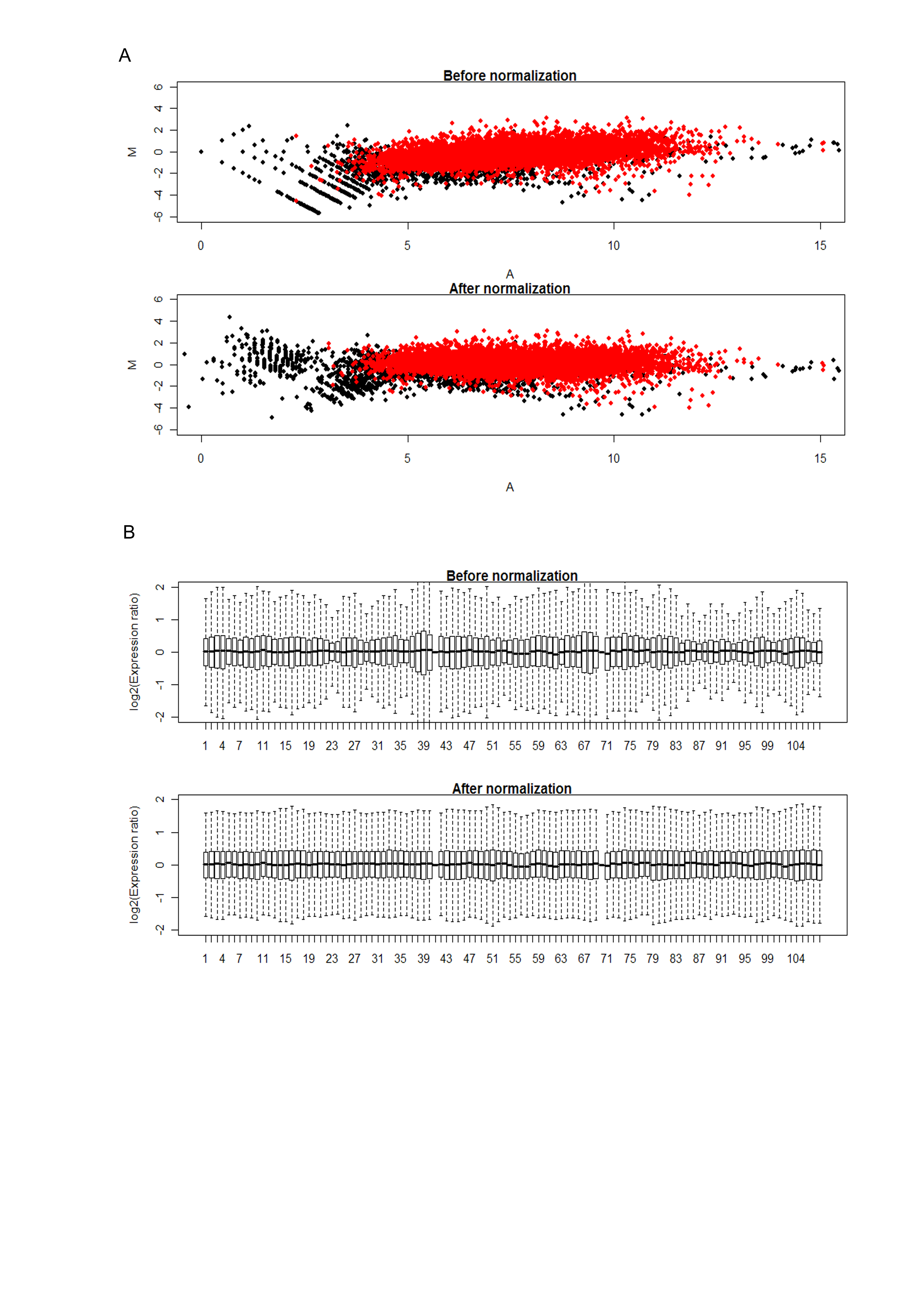

Supplement: Figure S3 — Quality Control and Normalizations. A. Expression ratio (y-axis) vs. mean intensity (x-axis) plots from 1 array on one field isolate before and after the “normexp” normalization and “robustspline” within-array normalization procedures were applied [123],[124]. Data points shown in black are those that were filtered out due to poor quality spots or screened out because they coded for VSAs or non-messenger RNA species. Data points shown in red are those probes included in the final analysis. B. Box-plots of data from each of 108 arrays before and after between-array normalization using the “scale” method in Limma [124]. Arrays 41 and 70 were excluded from analyses due to poor quality hybridizations. Slides 85–96 are for strain 3D7 (6 time-points, 2 replicates each). (5.38 MB TIF) [file ppat.1000644.s003.tif]

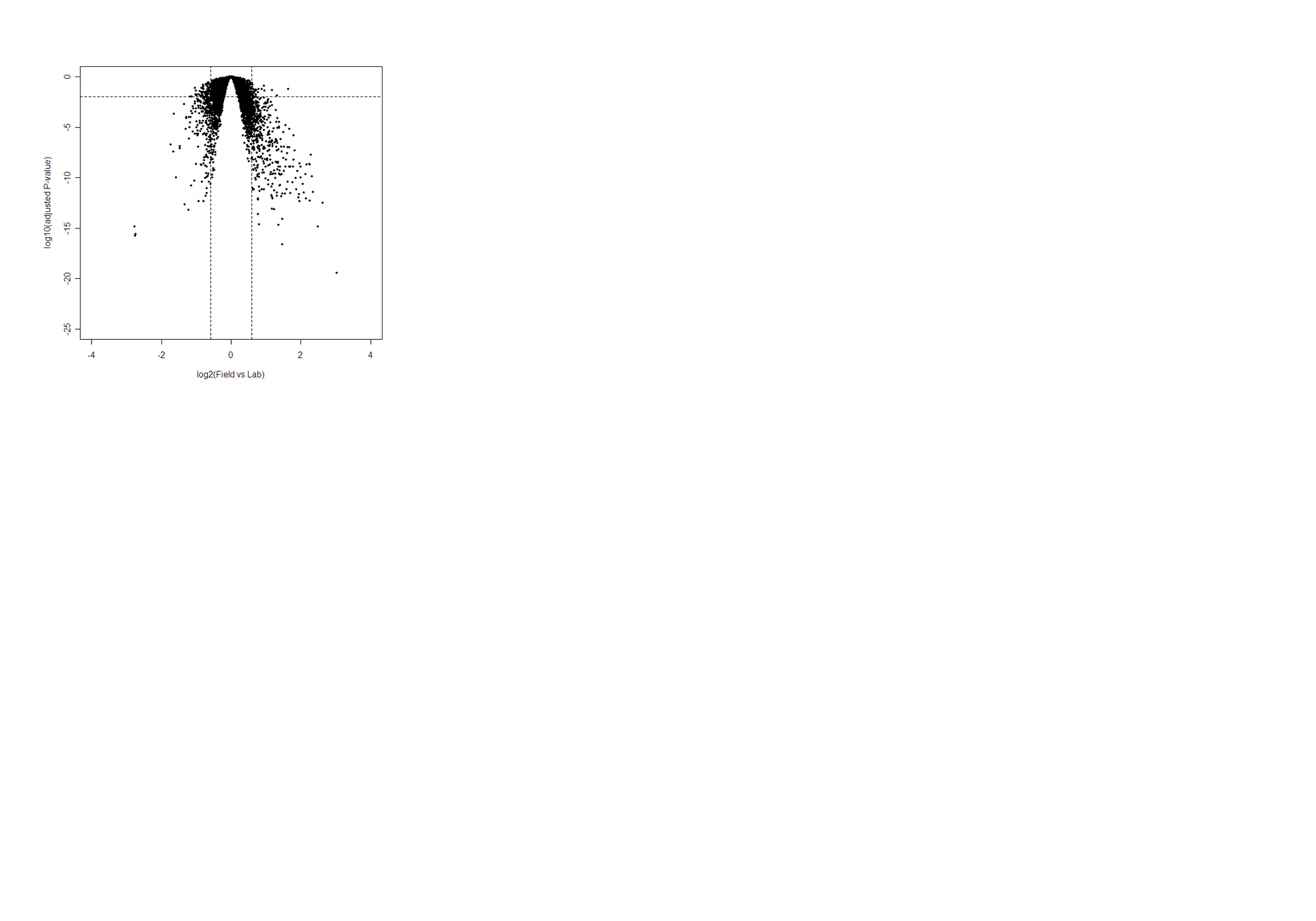

Supplement: Figure S4 — Volcano Plot Showing Relationship Between Expression Differences and P-values in Comparison of Field vs. Laboratory Isolates. Individual points are for probes and there are multiple probes per gene. The significance criteria of 1.5-fold expression and P<0.01 are indicated by the vertical and horizontal solid dashed lines, respectively, not allowing for multiple testing. After allowing for multiple testing, 6.8% (647/9493) of probes were significant. After including only genes for which the majority of their probes were significant, 259/4896 (5.3%) of genes were declared significant. (0.57 MB TIF) [file ppat.1000644.s004.tif]

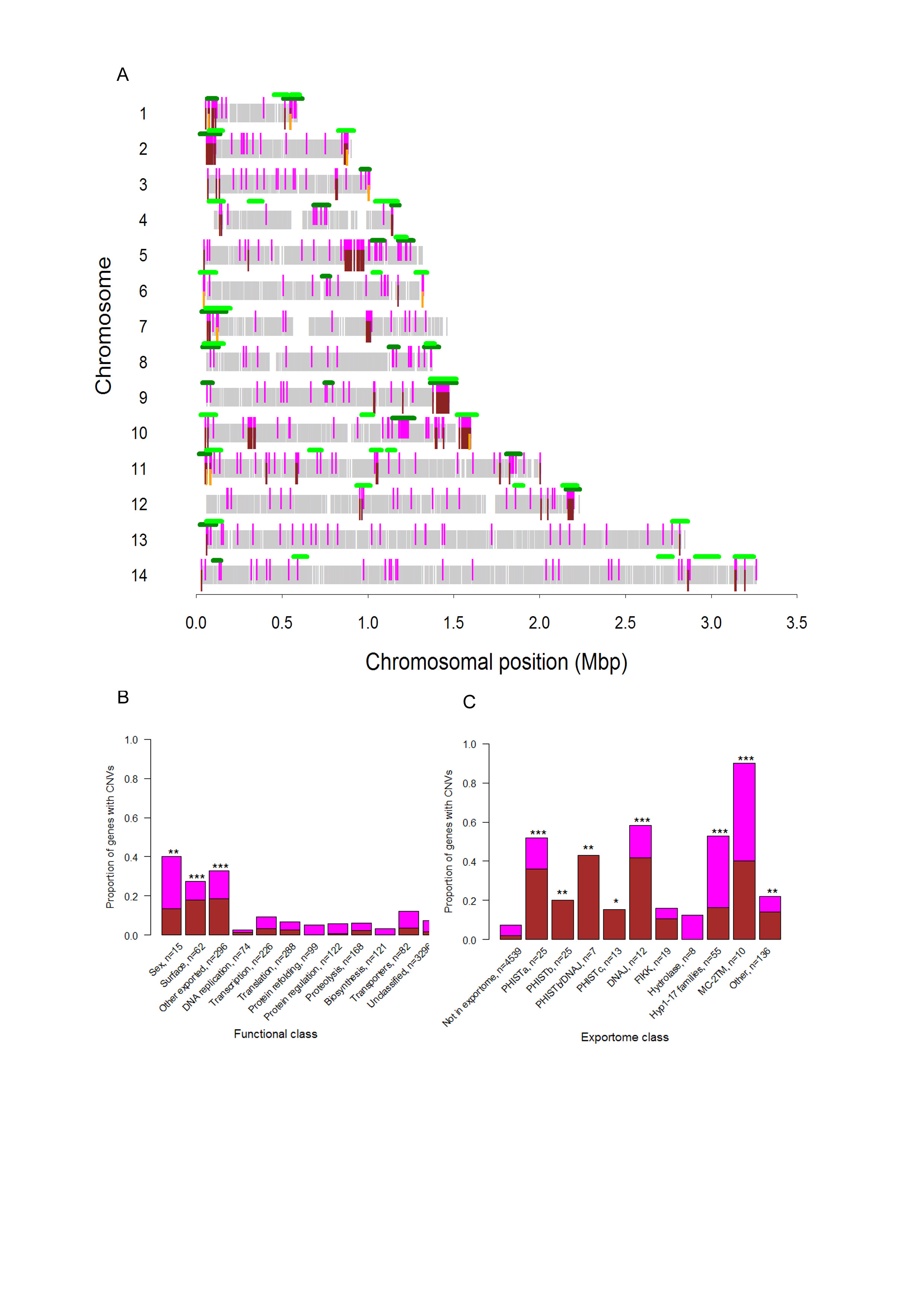

Supplement: Figure S5 — Chromosomal Location of CNVs and SDs and Functional Classification of Genes Contained Within Them. A. Chromosomal locations of genes that had significant variation among field isolates (P<0.01, >1.5-fold difference in at least two field isolates and the majority of their probes significant by these criteria) in genomic DNA content as measured by CGH. Pink bars indicate those CNVs detected in this study and brown bars indicate CNVs reported in previous studies (Table S2). Orange bars indicate the locations of SDs. Regions in which there were significantly more (P<0.05) CNVs than expected by chance (i.e., clustering) are indicated by a dark green bar above the chromosome. Clusters found in expression data (Figure 3) are shown in light green for comparison. Vertical grey bars indicate the locations of genes analyzed in this study. B. Genes were classified according to function based on their PlasmoDB annotations and GO terms, and bioinformatics studies (Table S1). The proportion of each class that were located in CNVs are shown for new CNVs found in this study (pink) and CNVs reported from previous studies (brown). C. As for B but classified according to their class within the exportome [57]. Numbers per class are shown in the x-axis labels. Significance levels for differences in proportions of each class vs. all other classes using the hypergeometric test are indicated by *, P<0.05; **, P<0.01; ***, P<0.001. (1.90 MB TIF) [file ppat.1000644.s005.tif]

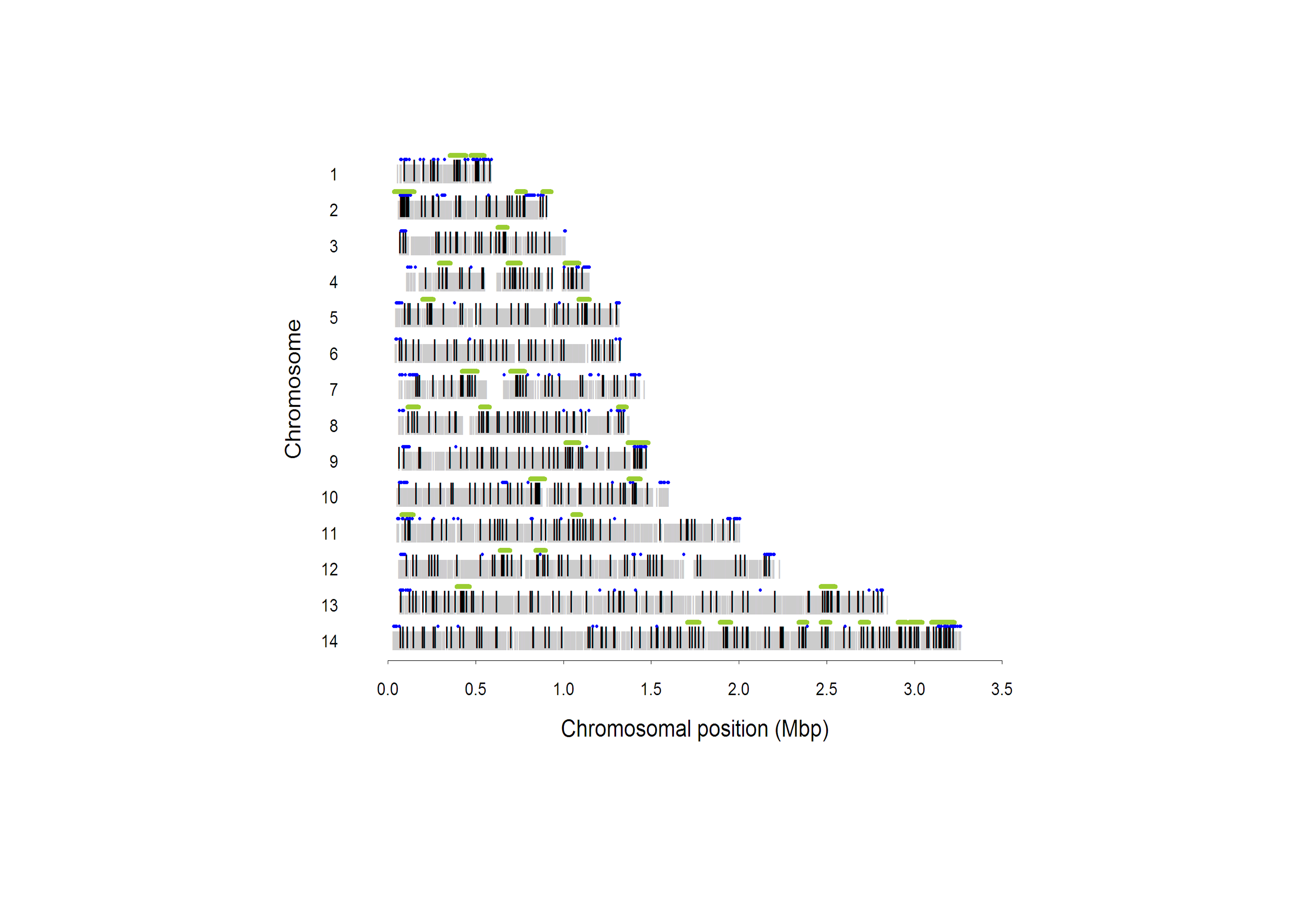

Supplement: Figure S7 — Chromosomal Location of Genes Differentially Expressed within Field Strains. Chromosomal locations of genes that showed significant variation (P<0.001 by F-test after correcting for multiple comparisons and which had the majority of their probes significant) in expression levels among field strains (black bars). Regions in which there were significantly more (P<0.05) differentially regulated genes than expected by chance (i.e., clustering) are indicated by a yellow-green bar above the chromosome. Genes belonging to the exportome are indicated by blue dots above the chromosome. Vertical grey bars indicate the locations of genes included in these analyses. (1.70 MB TIF) [file ppat.1000644.s007.tif]

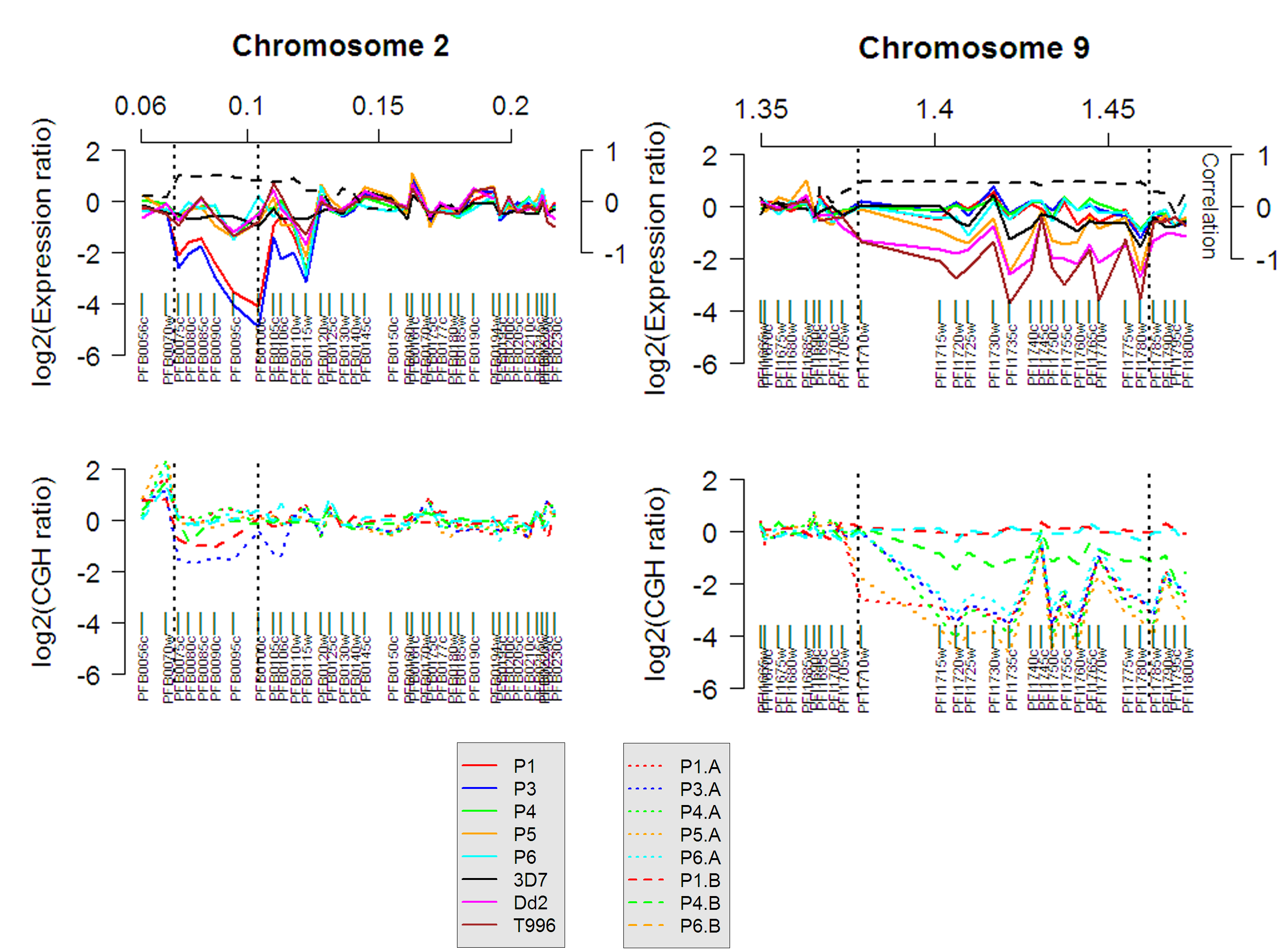

Supplement: Figure S8 — Gene Expression Levels and Genomic DNA Content for Each Parasite Isolate for Genes in the Region of the Chromosome 2 and 9 Deletions. Expression levels (left axis, upper panel) are expressed as differences from the mean over all time points for strain 3D7. Genomic content levels (lower panel) are expressed relative to 3D7 and are shown only for field isolates. The vertical dotted line indicates the site of the breakpoint as reported in previous studies. For Chromosome 2, based on expression levels, the deletion appears to have occurred in strains P1 and P3 beginning at PFB0100c (kharp) or PFB0105c as reported previously (marked by a vertical dashed line) and extends through to PFB0075c. This was confirmed by CGH in sub-lines P1.A and P3.A. In Chromosome 9, from the expression data, the laboratory strains Dd2 and T9/96 appear to have a deletion between genes PFI1710w (bporf) and PF1780w, consistent with previous reports of the breakpoint [41]. Strain P5 appears to be carrying a mixture of deleted and non-deleted parasites. These breakpoints were confirmed in CGH analysis of the field strains, all of which carried the deletion in at least one sub-line after further culturing. In the expression data (upper panel), the black dashed line shows the across-strain correlations (right axis) between expression profiles for an index gene inside the deleted region (PFB0105c or PFI1780w) and other genes in the region. The correlations remain positive beyond the deletion boundaries, indicating co-regulation of genes neighboring the deletion. Two strongly down-regulated genes adjacent to the deletion boundaries, PFB0115w and PFI1785w, have been shown in previous studies to be up-regulated in parasites obtained from pregnant women (Table S3). (2.45 MB TIF) [file ppat.1000644.s008.tif]

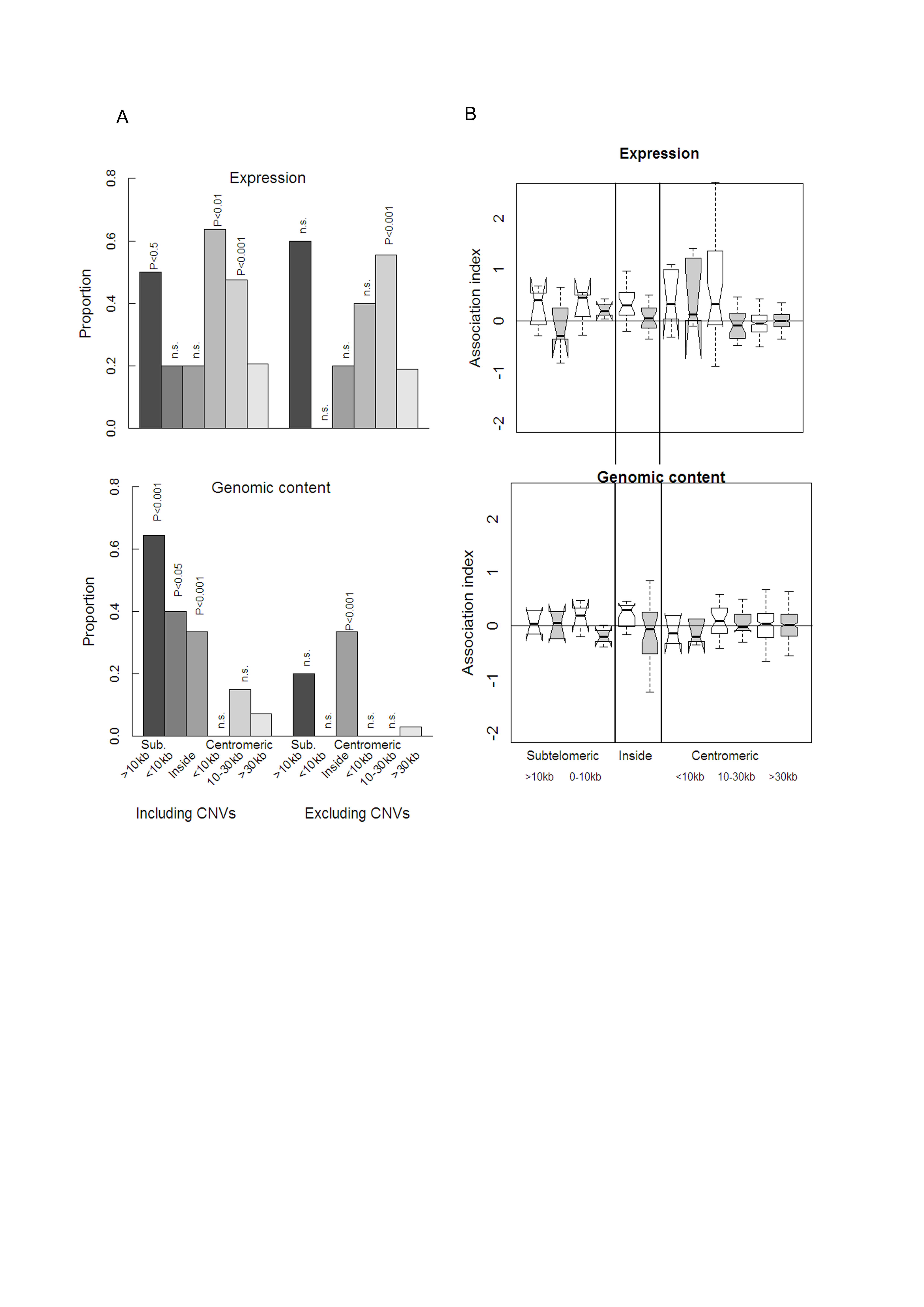

Supplement: Figure S9 — Co-regulation of Genes Surrounding SDs. A. Proportion of genes significantly differentially regulated between field and laboratory isolates or among field isolates (upper) and showing significant between-strain variation at the genomic level (lower) grouped by distance of the gene from the SD boundary on either its subtelomeric side or centromeric side. P-values (by Fisher's Exact test) indicate where the proportion of significant genes was different from that >30kb centromeric to the SD boundary (right-hand bar in each group of bars). Results are shown with (left) and without (right) data from genes contained within CNVs that are not also SDs. B. Across-strain (field and laboratory strains) measures of association (regression coefficients, white bars) between expression levels (upper) of a single gene within a SD (the index gene, hyp4, or for SD11LA, gene PF11_0015, not necessarily differentially expressed) and expression levels of genes surrounding the SD (test gene, not necessarily differentially expressed) grouped by distance from the SD boundary. The same analysis was performed for genomic content data (lower). To compare these results with those expected by chance, associations after randomly permuting the data across strains within genes were computed (grey bars). The horizontal line inside each box shows the median, the box boundaries show the interquartile range, the whisker length is one interquartile range, the box width is proportional to the square root of the number of observations per group, and the notches show the approximate 95% confidence intervals, i.e., non-overlapping notches strongly support a hypothesis of non-equivalence [135]. Regression analysis of the data from individual genes, using only gene that were differentially expressed in order to rule out bias due to higher numbers of significant genes nearer to SDs, showed a significant decline in the strength of association with distance from the SD boundary (P<0.001). This decline was strong [file ppat.1000644.s009.tif]

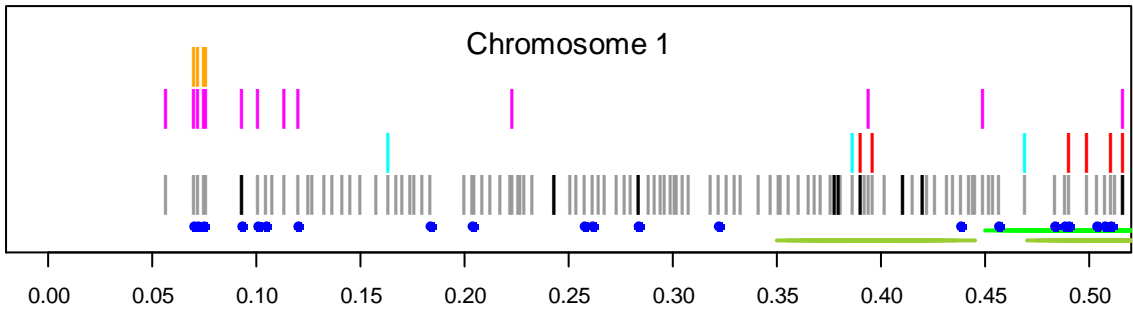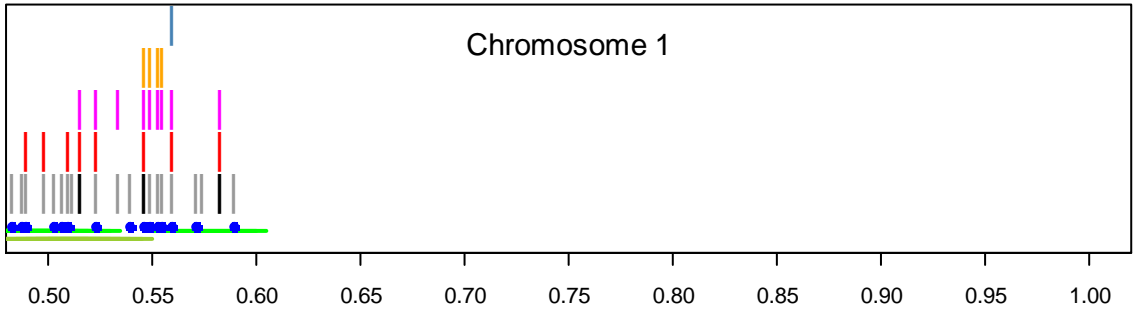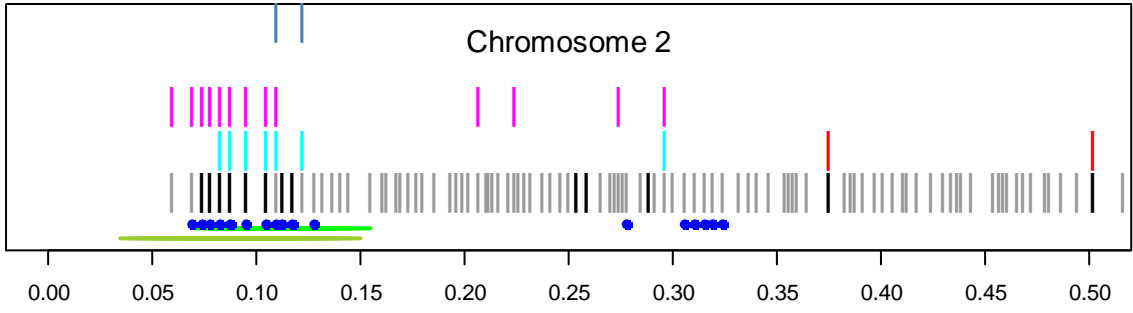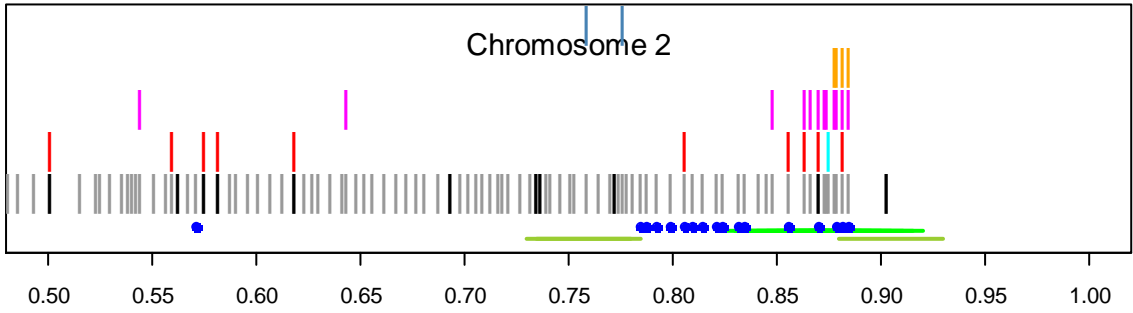

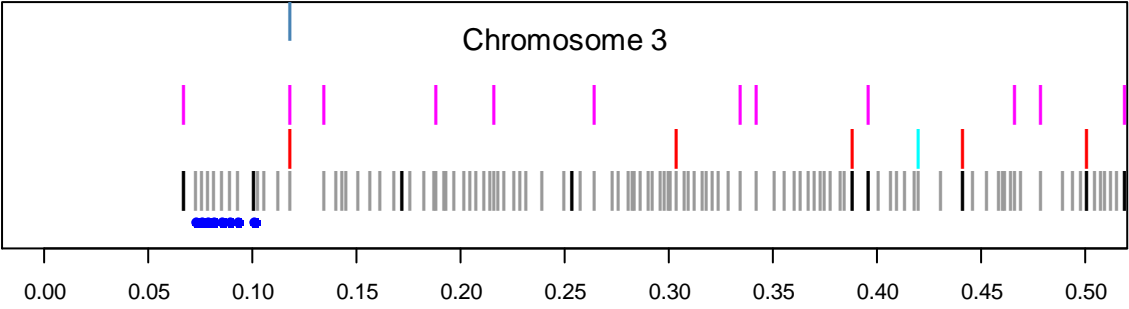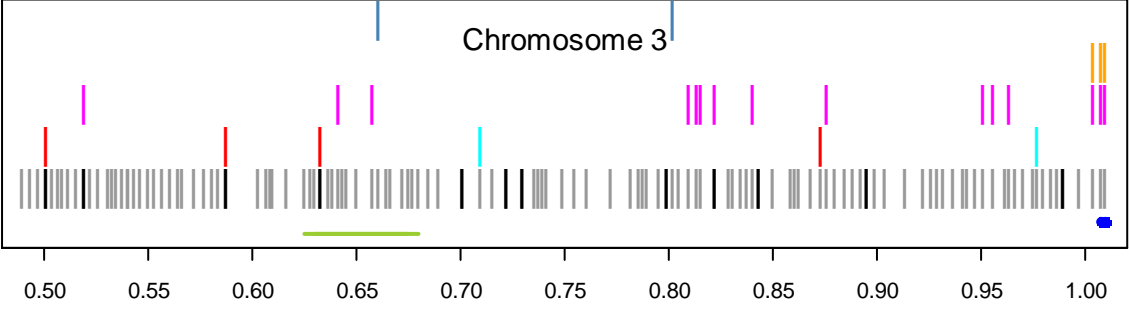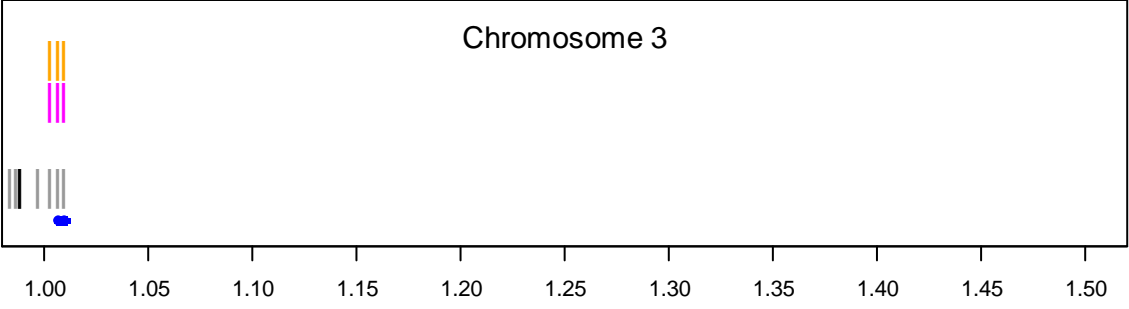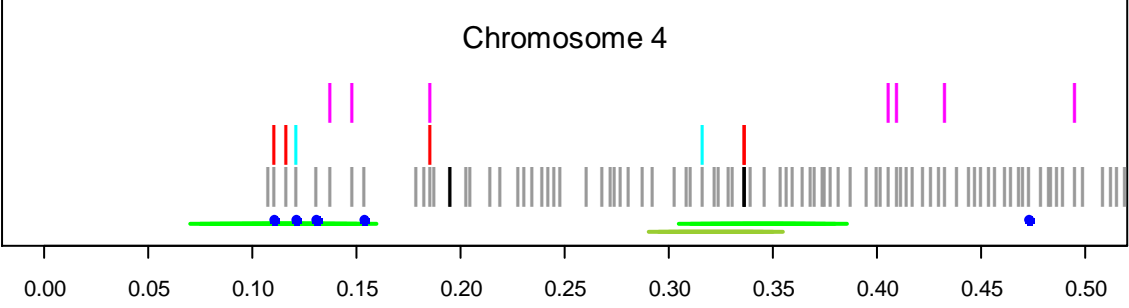

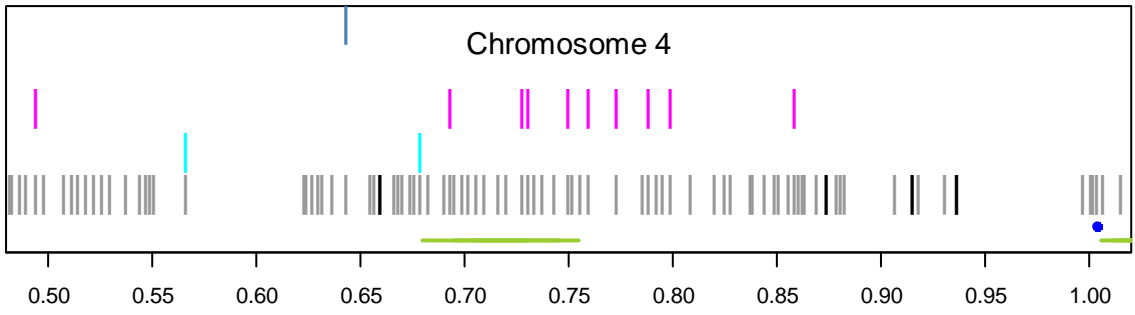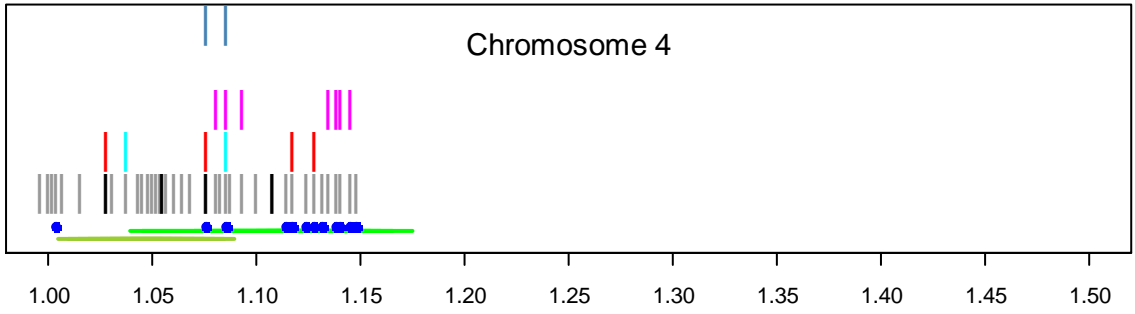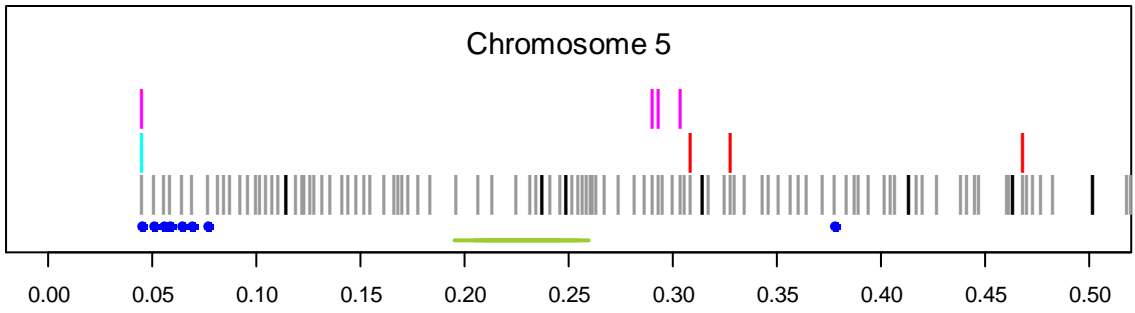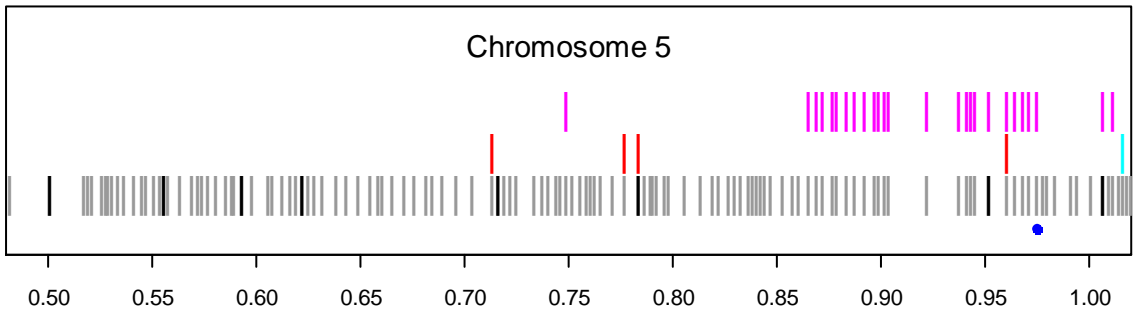

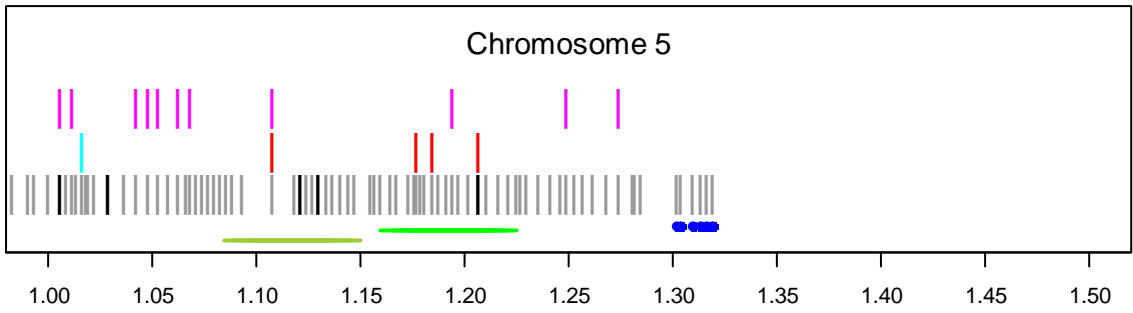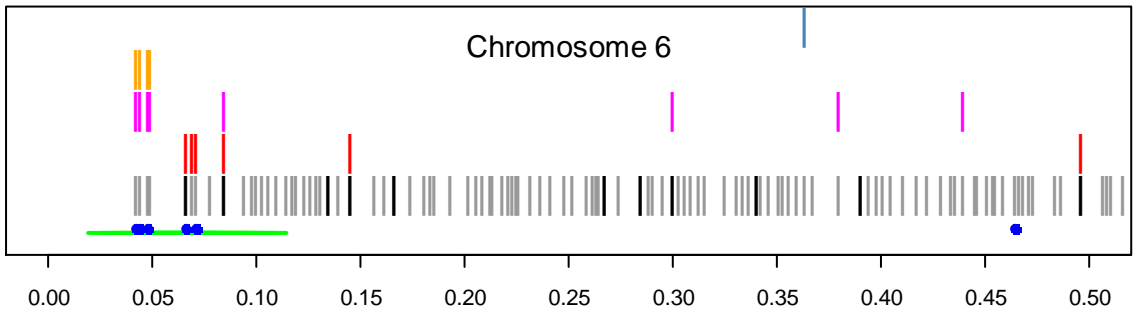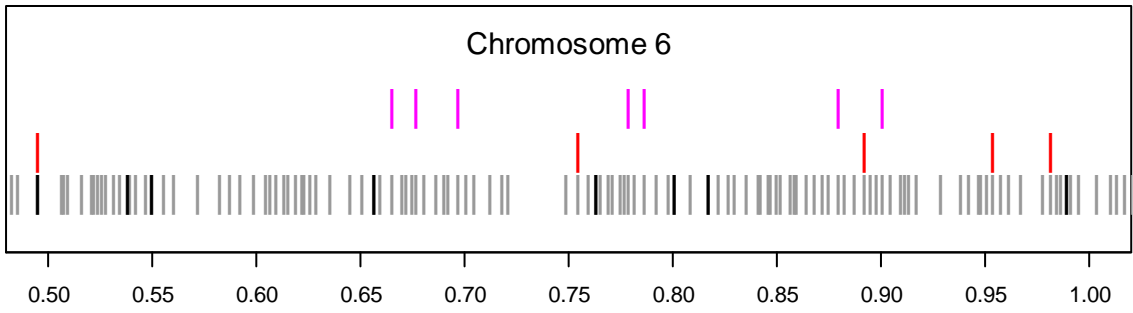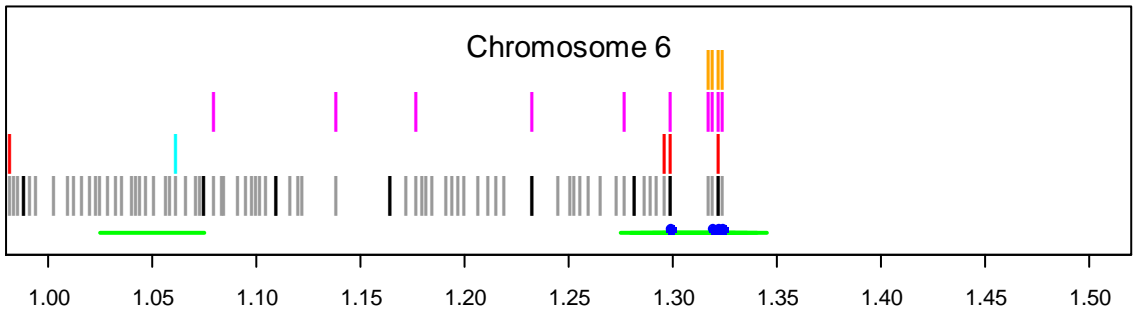

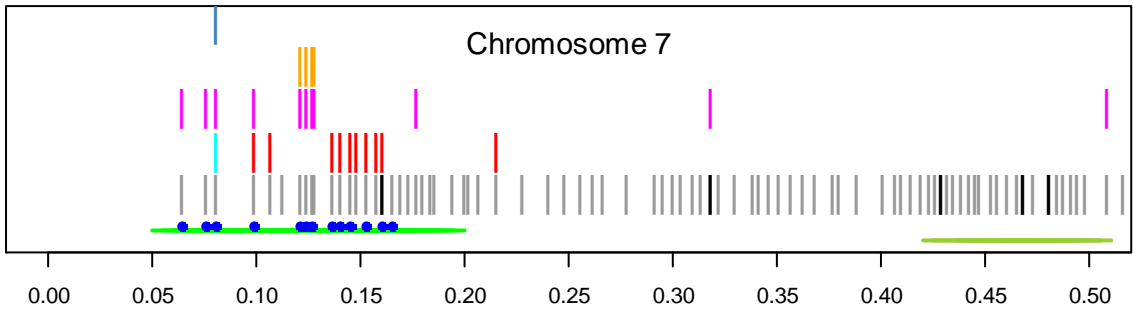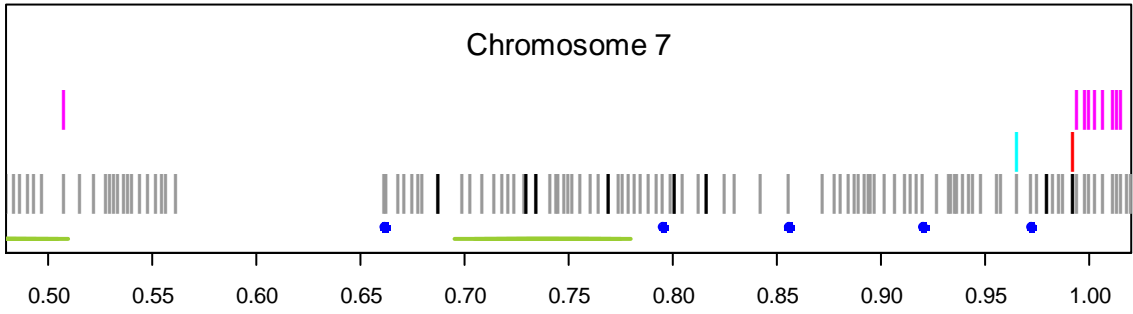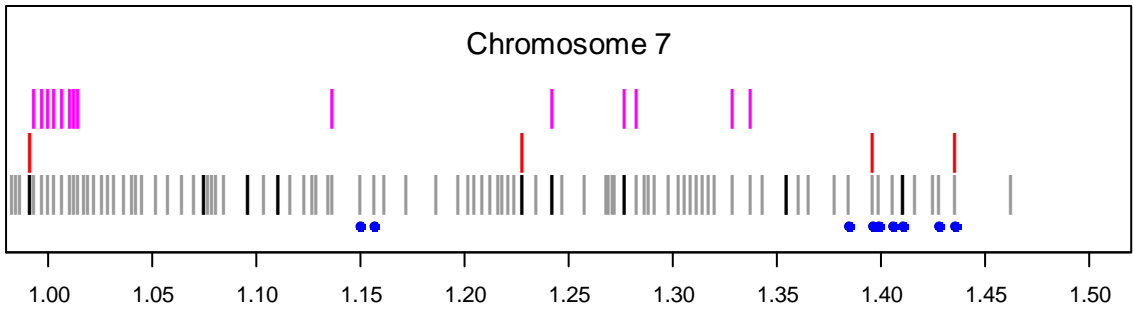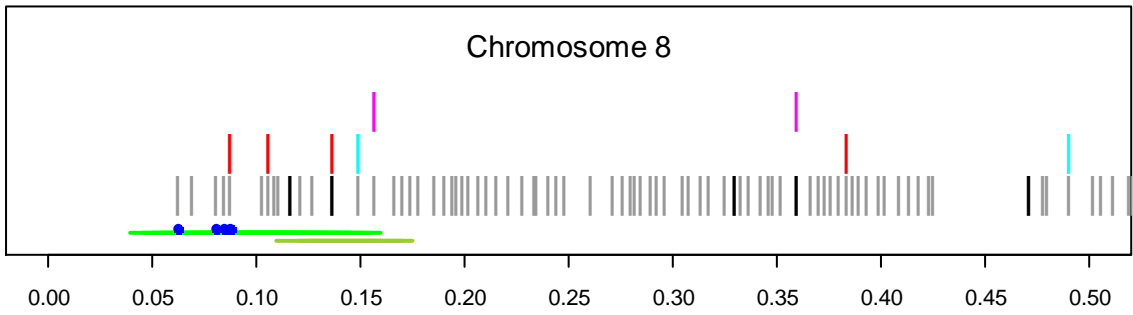

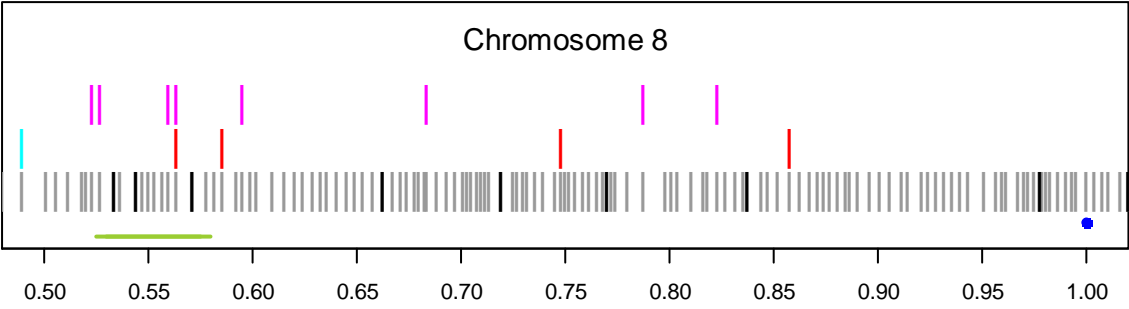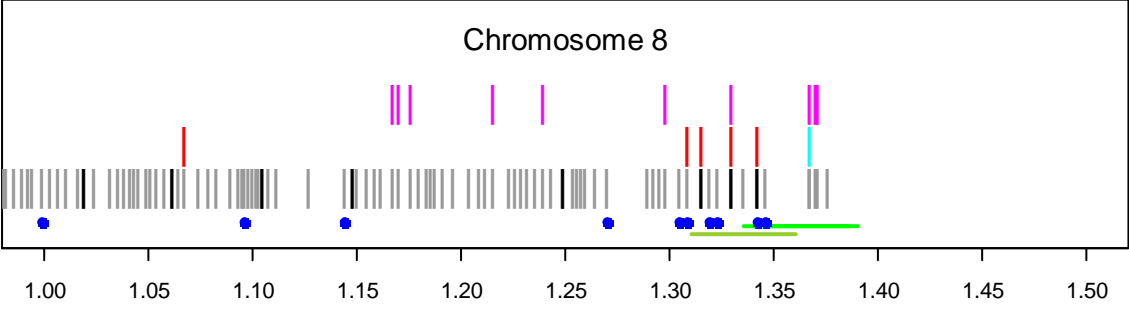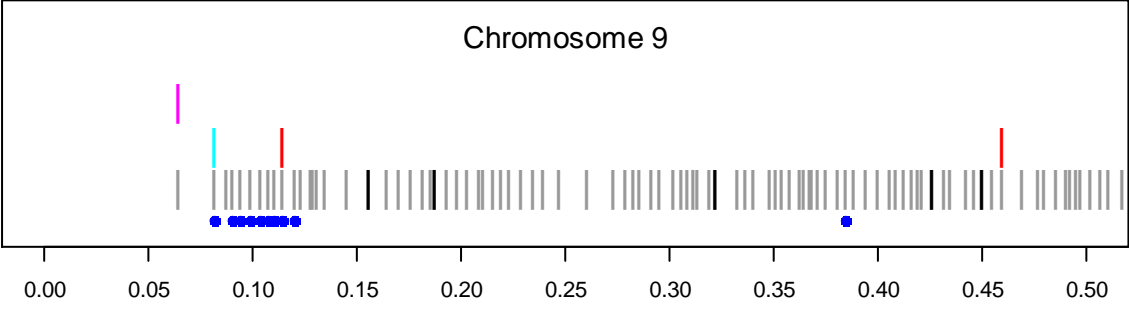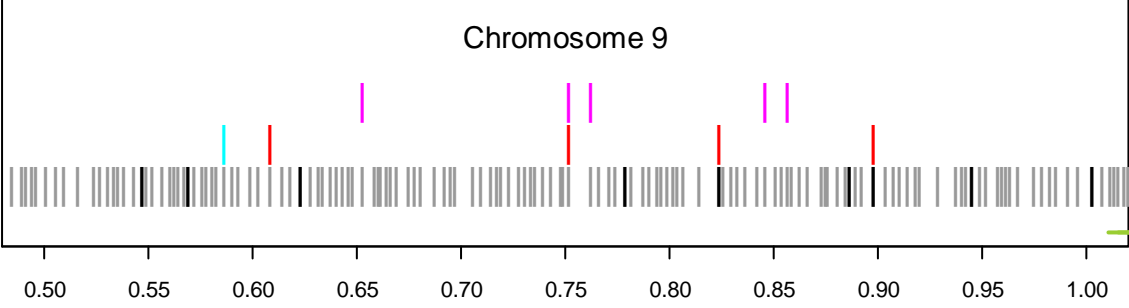

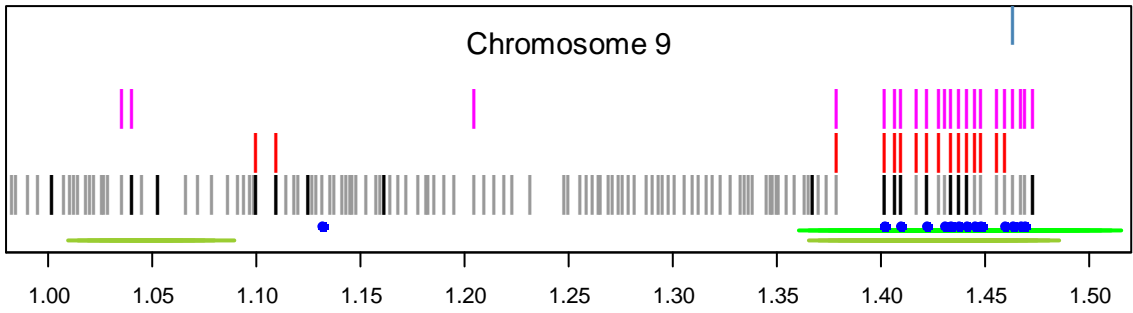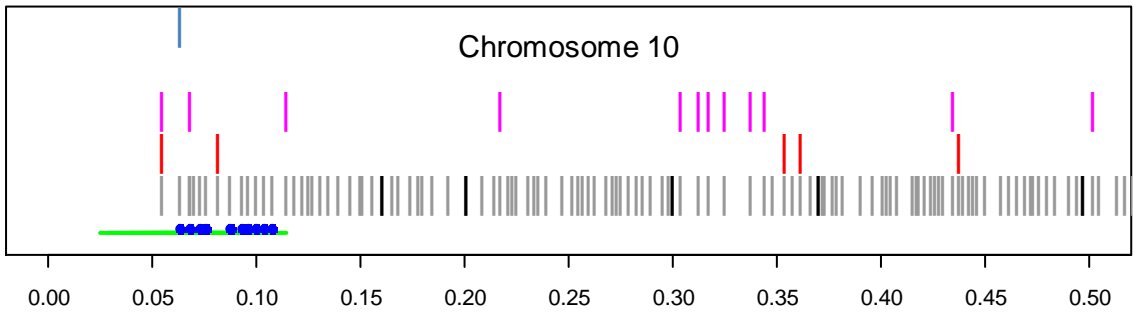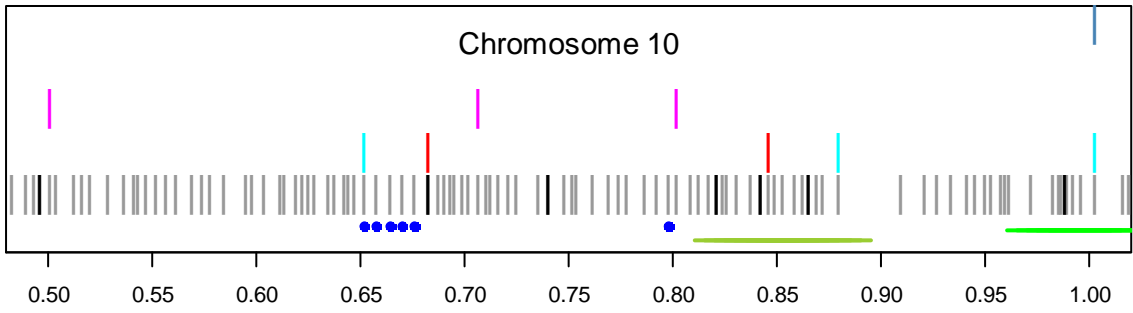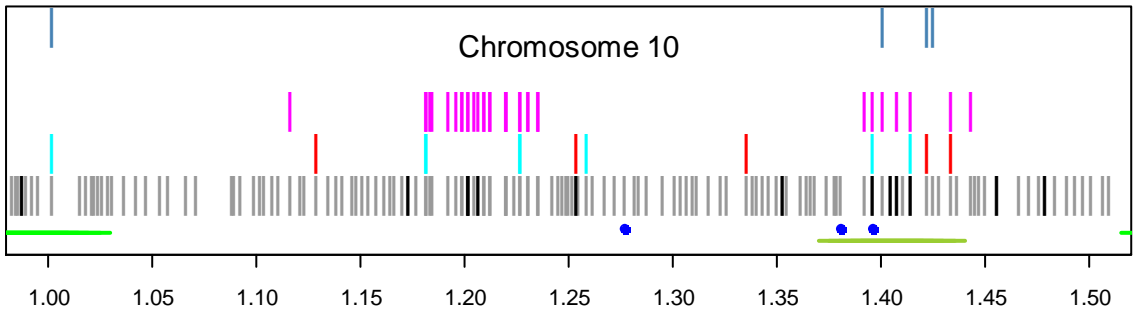

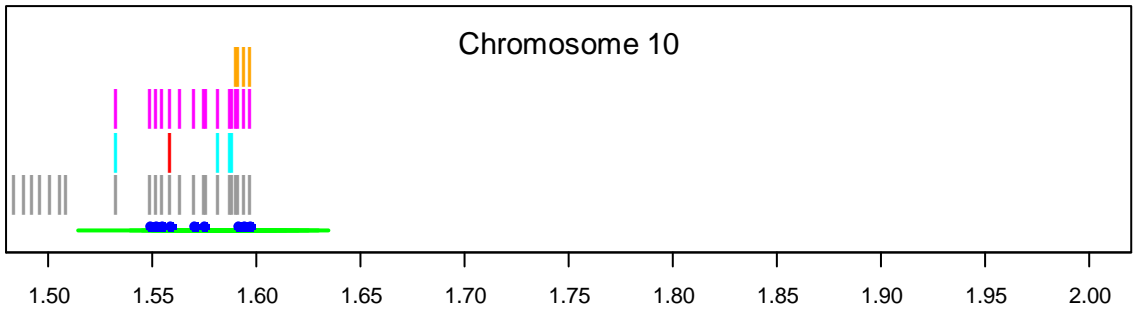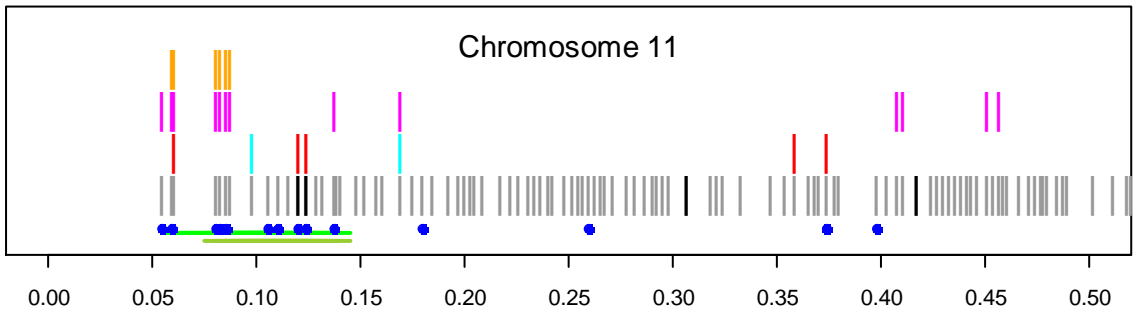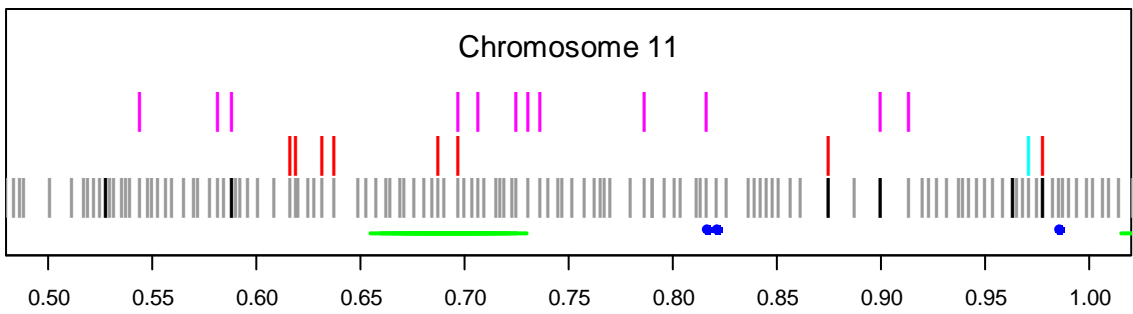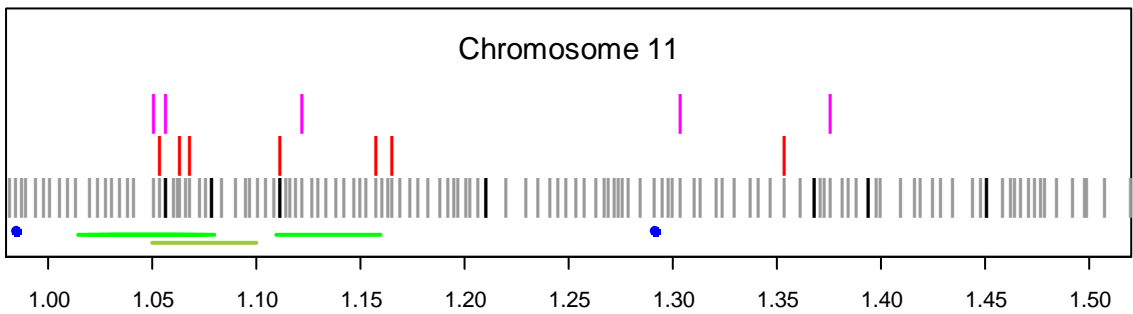

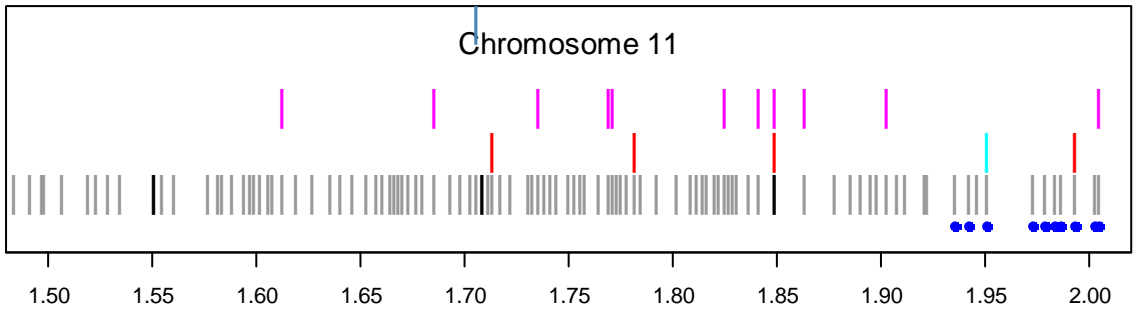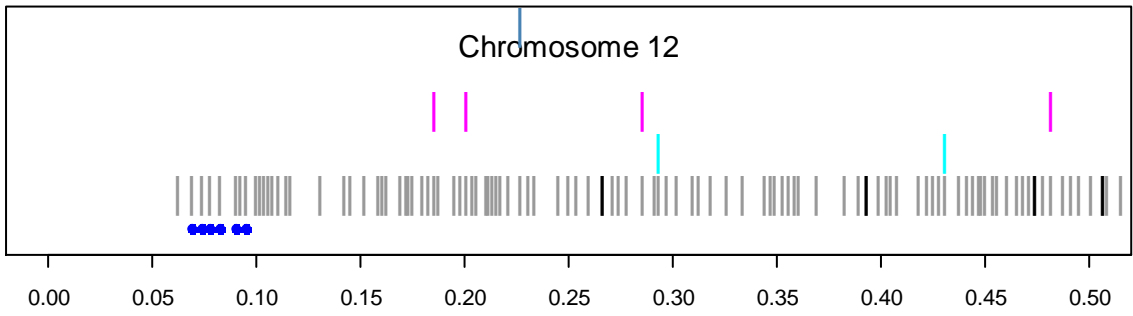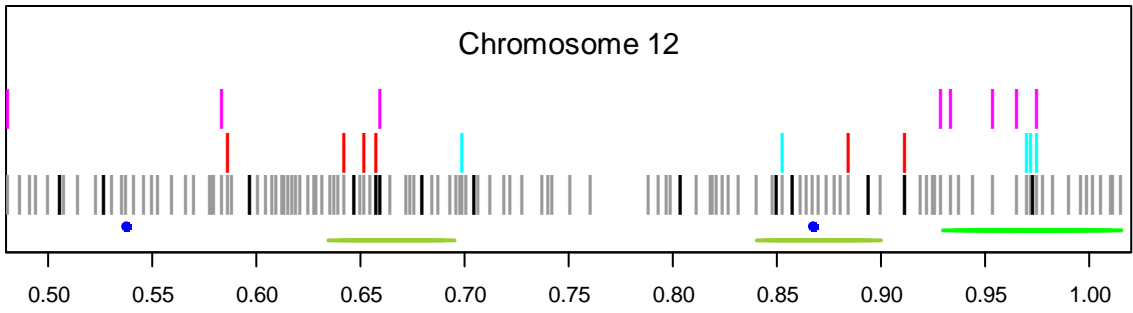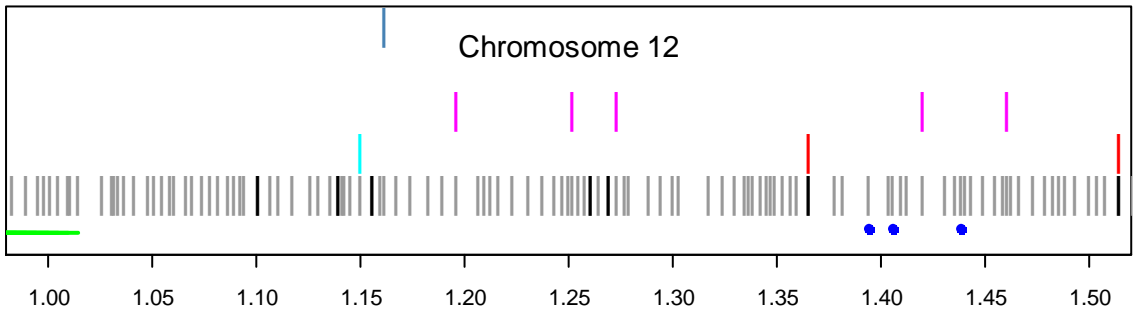

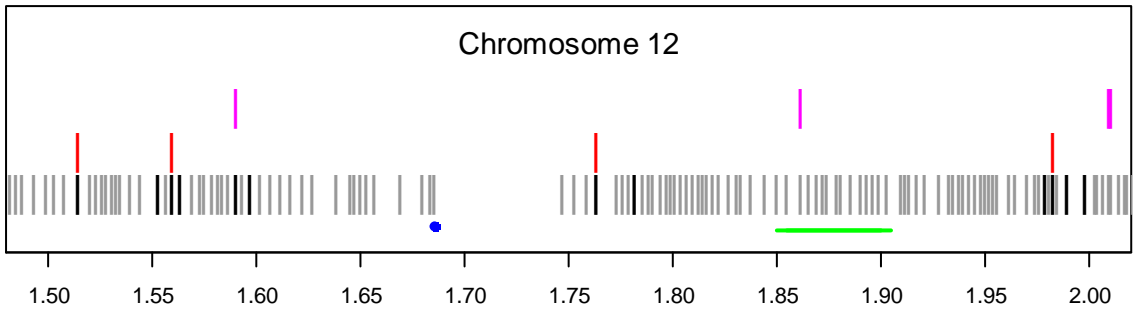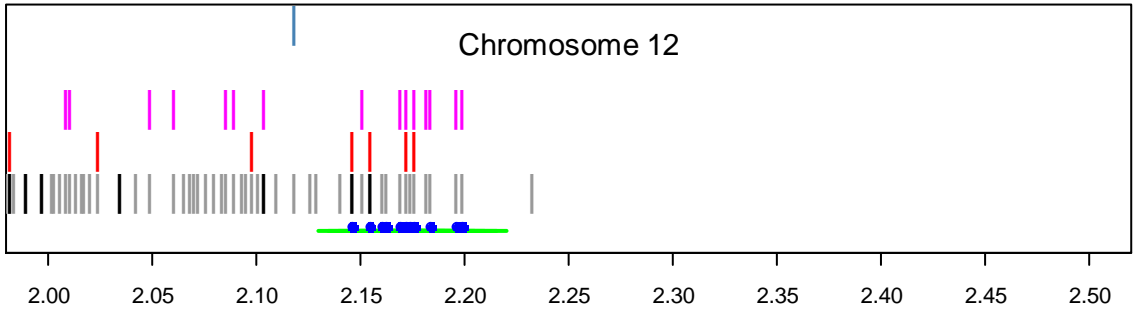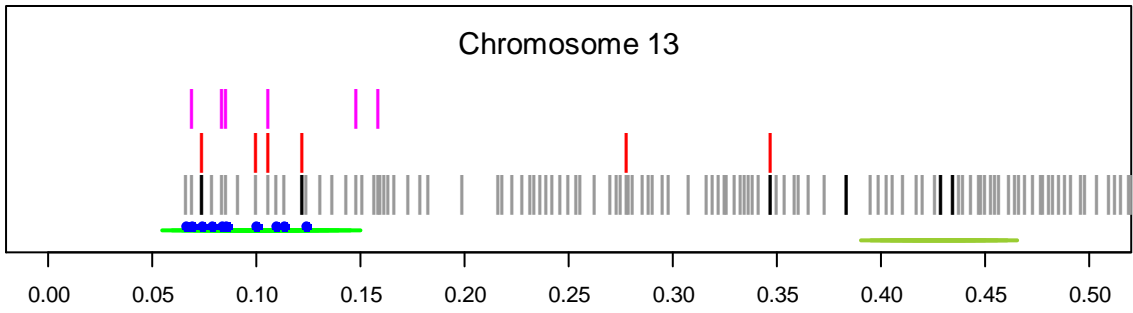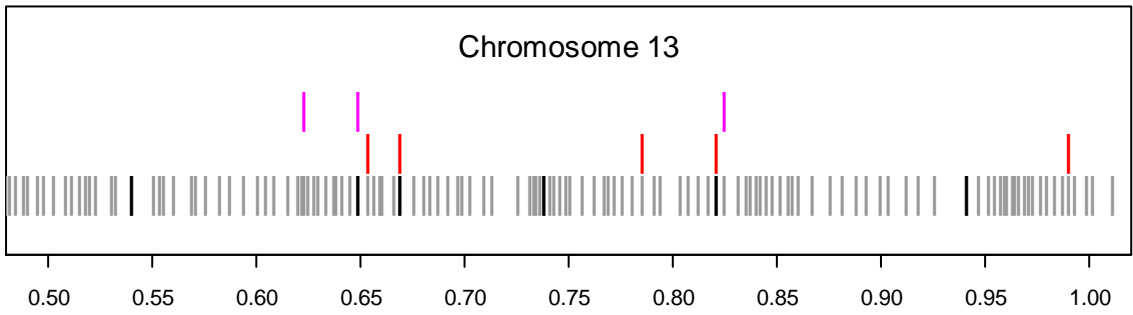

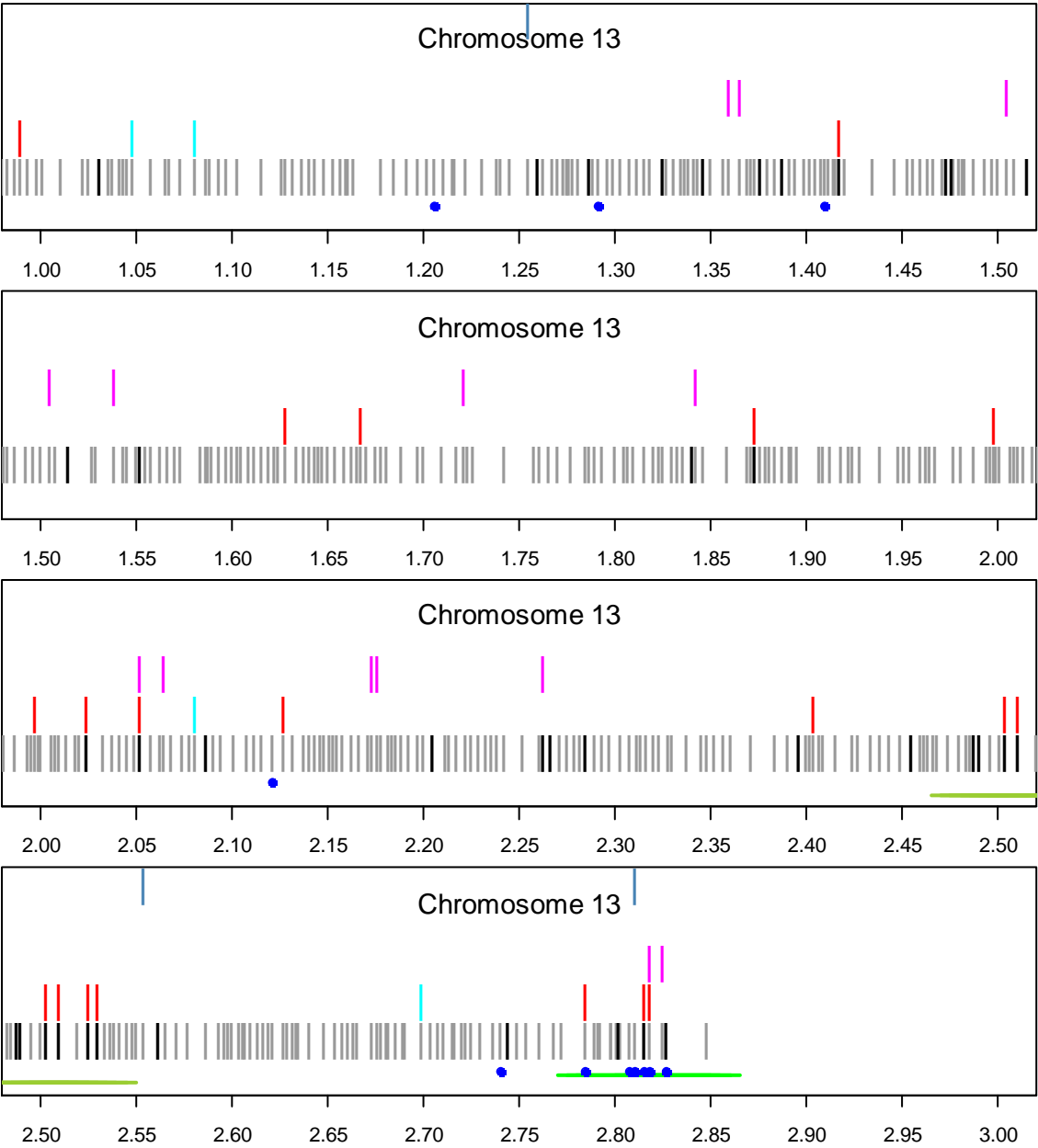

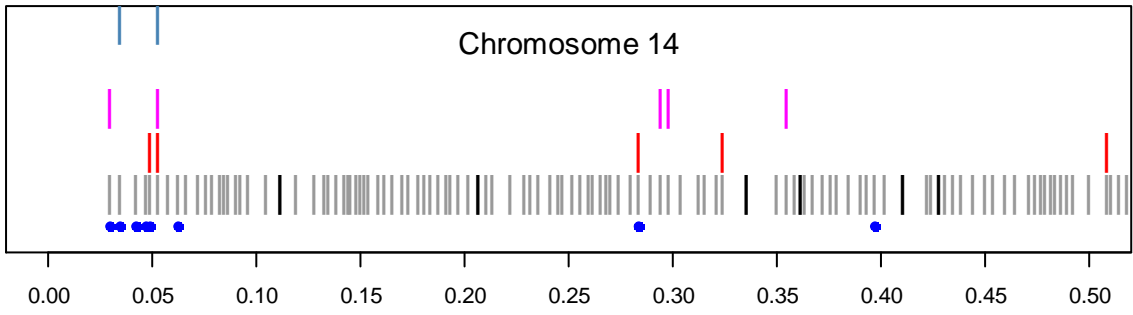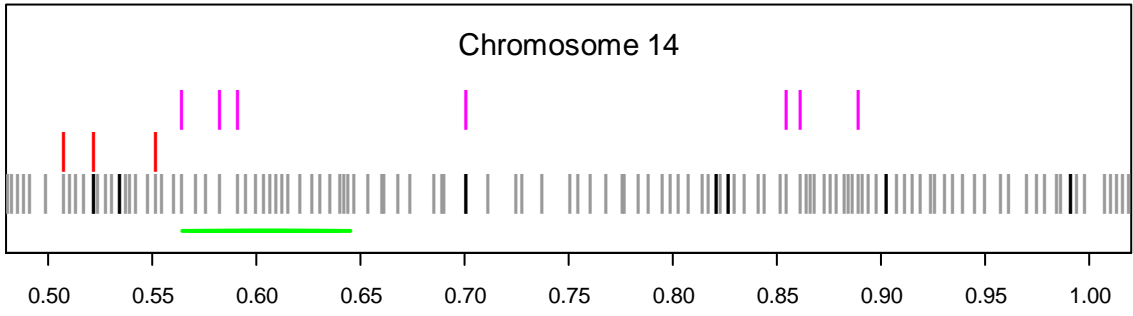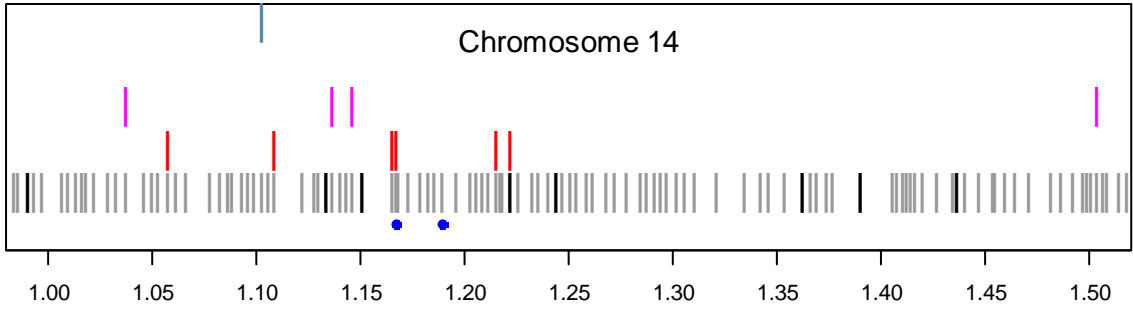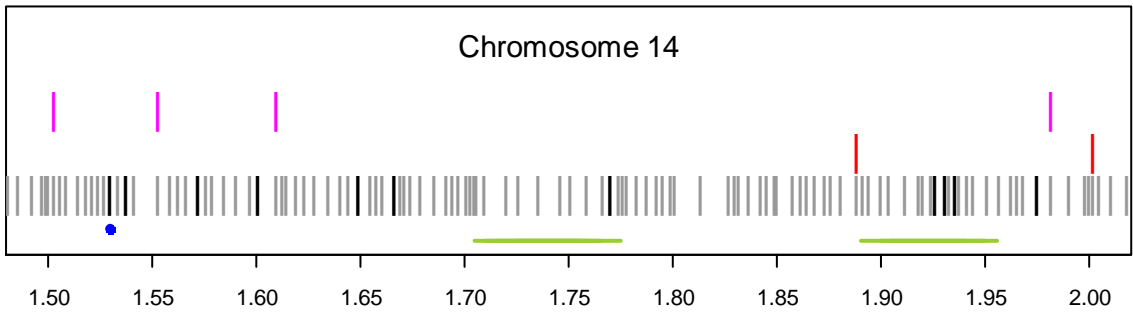

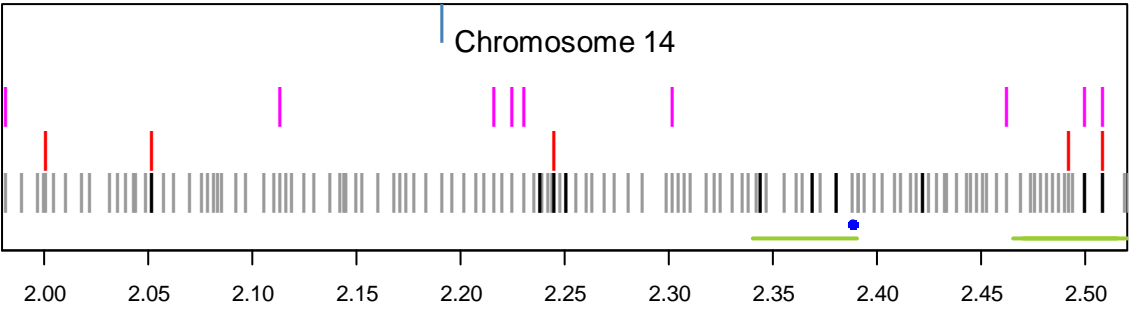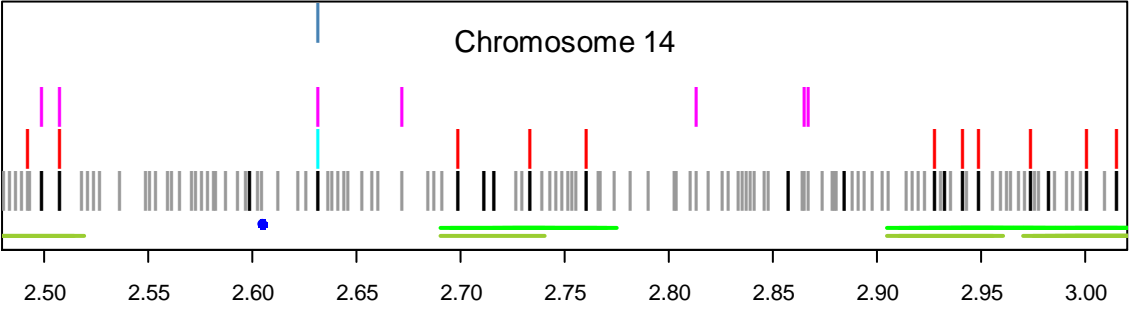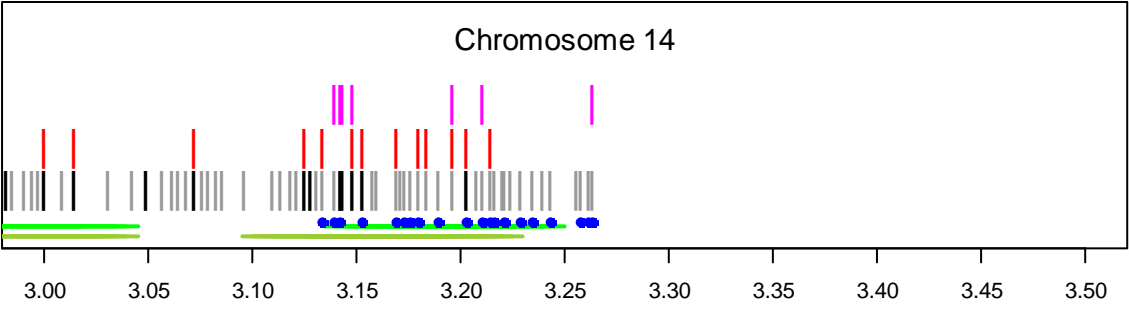

Supplement: Figure S10 — Regional Genomic Maps of CNVs, SDs, Differentially Expressed Genes, Expression Clusters, CNV Clusters and Pregnancy-Associated Genes from This and Other Studies. Grey bars indicate the locations of those genes analysed in this study. Superimposed black bars mark those genes differentially regulated within field isolates. In the next row up, red and light blue bars indicate genes up- and down-regulated genes in field vs. laboratory isolates, respectively. Pink bars in the third row mark CNVs found in this or other studies. In the fourth row orange bars represent SDs. Steel blue bars in the fifth row up show genes reported to be associated with placental malaria. Blue dots below the first row indicate exportome genes. Green horizontal lines marks regions where significant clustering (P<0.05) of genes differentially expressed between field and laboratory isolates were found, while yellow-green lines indicate significant clustering of CNVs. (0.14 MB PDF) [file ppat.1000644.s010.pdf]
