# Supplementary material for: Comparative Transcriptional and Genomic Analysis of Plasmodium falciparum Field Isolates
Source: PLoS Pathog. 2009 Oct 30;5(10):e1000644. doi: 10.1371/journal.ppat.1000644 (PMC2764095; doi:10.1371/journal.ppat.1000644)
Supplement: Table S1 — Terms used for classifying genes according to function. String searches were applied to the fields of Product Description, GO Annotated Function, GO Annotated Process and GO Annotated Component downloaded from the list of updated annotations in PlasmoDB version 5.5. (0.05 MB DOC) [file ppat.1000644.s011.doc]

**Table S1.** Terms used for classifying genes according to function. String searches were applied to the fields of Product Description, GO Annotated Function, GO Annotated Process and GO Annotated Component downloaded from the list of updated annotations in PlasmoDB version 5.5.

| **Classification** | **Terms searched for** |
| --- | --- |
| **DNA replication** | DNA replication, replication origin, DNA repair |
| **Transcription** | transcription, RNA splicing, zinc-finger, RNA metabolism |
| **Translation** | translation, ribosome, RNA modification |
| **Protein refolding** | chaperone, chaperonin, heat shock,protein folding, refolding |
| **Protein regulation** | protein kinase, phosphatase, GTP-binding, GTPase, Ras and those listed in the kinome [1] |
| **Proteolysis** | proteolysis, proteinase, protease, ubiquitin |
| **Biosynthesis** | Biosynthesis |
| **Transporters** | Those listed in the permeome [2] |
| **Sex** | gametocyte, gamete, sexual, transmission, ookinete, sporozoite, male, female, zygote |
| **Surface** | cytoadherence, surface, exported protein, and all those in Cooke 2004 (excluding those included in Sex) |
| **Other exported** | Those listed in Sargeant et al. (2006) [3] and not in Sex or Surface |
| **Not classified** | None of the above |
| **Unknown** | hypothetical protein, unknown function |

References

1. Ward P, Equinet L, Packer J, Doerig C (2004) Protein kinases of the human malaria parasite Plasmodium falciparum: the kinome of a divergent eukaryote. BMC Genomics 5: 79.

2. Martin RE, Henry RI, Abbey JL, Clements JD, Kirk K (2005) The 'permeome' of the malaria parasite: an overview of the membrane transport proteins of *Plasmodium falciparum*. Genome Biol 6: R26.

3. Sargeant TJ, Marti M, Caler E, Carlton JMR, Simpson K, Speed TP, Cowman AF (2006) Lineage-specific expansion of proteins exported to erythrocytes in malaria parasites. Genome Biol 7: R12.
