# Supplementary material for: Comparative Transcriptional and Genomic Analysis of Plasmodium falciparum Field Isolates
Source: PLoS Pathog. 2009 Oct 30;5(10):e1000644. doi: 10.1371/journal.ppat.1000644 (PMC2764095; doi:10.1371/journal.ppat.1000644)
Supplement: Table S2 — CNVs and SDs identified in this and other studies. (0.82 MB PDF) [file ppat.1000644.s012.pdf]

Table S2. CNVs and SDs identified in this and other studies.

[a]Name of CNV	[a]Name of SD	[b]Gene ID	Chromosome	Bp at gene midpoint	[c]Kidgell	[c]Ribacke	[c]Bozdech	[c]Carret	[d]Others	[e]This study
CNV1A		PFA0035c	1	57015				*	Pologe1990	0
CNV1A	SD1L	PFA0055c	1	70094					Pologe1990	0
CNV1A	SD1L	PFA0060w	1	72258					Pologe1990	0
CNV1A	SD1L	PFA0065w	1	75198					Pologe1990	0
CNV1A	SD1L	PFA0070c	1	76629					Pologe1990	0
CNV1A		PFA0100c	1	93706					Pologe1990	-2
CNV1A		PFA0110w	1	100784		*			Pologe1990	0
CNV1A		PFA0115w	1	105189						+/-2
CNV1A		PFA0125c	1	113509				*		0
CNV1A		PFA0130c	1	120462						2
CNV1B		PFA0170c	1	150580						3
CNV1C		PFA0200w	1	176228						-2
CNV1D		PFA0500w	1	393974						-3
CNV1E		PFA0650w	1	516120		*		*		-4
CNV1F	SD1R	PFA0675w	1	546452						0
CNV1F	SD1R	PFA0680c	1	549711				*		-5
CNV1F	SD1R	PFA0685c	1	552867						-2
CNV1F	SD1R	PFA0690w	1	554941						1
CNV1F		PFA0700c	1	560177						5
CNV1F		PFA0715c	1	571600						+/-2
CNV1F		PFA0720w	1	574056						2
CNV1F		PFA0725w	1	582467						-2
CNV2A		PFB0056c	2	60213				*	Pologe1988, Scherf1992A	2

CNV2A	PFB0070w	2	69430	*	*	*	*	Pologe1988, Scherf1992A	5
CNV2A	PFB0075c	2	73919	*	*	*	*	Pologe1988, Scherf1992A	-2
CNV2A	PFB0080c	2	78030	*	*		*	Pologe1988, Scherf1992A	-3
CNV2A	PFB0085c	2	82728	*	*	*	*	Pologe1988, Scherf1992A	-2
CNV2A	PFB0090c	2	87733	*	*		*	Pologe1988, Scherf1992A	-2
CNV2A	PFB0095c	2	95078	*	*		*	Pologe1988, Scherf1992A	-2
CNV2A	PFB0100c	2	104591		*		*	Pologe1988, Scherf1992A	-1
CNV2A	PFB0105c	2	110072				*		-1
CNV2A	PFB0106c	2	113115						-1
CNV2B	PFB0210c	2	206646						-1
CNV2C	PFB0285c	2	265192						+3
CNV2D	PFB0300c	2	274098						-3
CNV2D	PFB0310c	2	277975						2
CNV2E	PFB0330c	2	295945						-5
CNV2F	PFB0365w	2	331568						-2
CNV2G	PFB0405w	2	375142						-3
CNV2H	PFB0570w	2	523465						-2
CNV2I	PFB0710c	2	643712						5
CNV2J	PFB0865w	2	753202						3
CNV2K	PFB0950w	2	848302						-2
CNV2L	PFB0960c	2	863612			*	*		0
CNV2M	PFB0965c	2	866119	*		*			0
CNV2M	PFB0970c	2	870314	*					0
CNV2M	PFB0972w	2	873223		*	*			0

CNV2M		PFB0973c	2	874012	*	*			0
CNV2N	SD2R	PFB0980w	2	877406					0
CNV2N	SD2R	PFB0985c	2	878858		*	*		0
CNV2N	SD2R	PFB0990c	2	881866					0
CNV2N	SD2R	PFB0995w	2	883924					0
CNV3A		PFC0050c	3	67366		*			0
CNV3B		PFC0110w	3	118777			*	Iriko2008	-7
CNV3B		PFC0120w	3	134718				Iriko2008	-7
CNV3C		PFC0205c	3	216169					-4
CNV3D		PFC0245c	3	264061					2
CNV3E		PFC0270w	3	290421					4
CNV3E		PFC0271c	3	292276					2
CNV3F		PFC0345w	3	355562					3
CNV3G		PFC0390w	3	395757					-3
CNV3H		PFC0460w	3	466379					-3
CNV3I		PFC0475c	3	478464					6
CNV3J		PFC0520w	3	519101					-3
CNV3K		PFC0582c	3	566721					1
CNV3L		PFC0595c	3	576678					-5
CNV3M		PFC0695w	3	641401					-2
CNV3N		PFC0865w	3	812882					3
CNV3N		PFC0870w	3	814974	*				2
CNV3N		PFC0875w	3	822425	*				0
CNV3O		PFC0925w	3	875293					5
CNV3P		PFC1016w	3	961324					2
CNV3Q		PFC1060c	3	989255					4
CNV3R	SD3R	PFC1075w	3	1003046					0
CNV3R	SD3R	PFC1080c	3	1004478					-1
CNV3R	SD3R	PFC1085c	3	1007411					0
CNV3R	SD3R	PFC1090w	3	1009580					0

CNV4A	PFD0100c	4	137547		*			0
CNV4A	PFD0110w	4	148605		*			0
CNV4B	PFD0150w	4	185818					7
CNV4C	PFD0403w	4	409295					7
CNV4D	PFD0735c	4	682802					3
CNV4E	PFD0745c	4	693710					3
CNV4F	PFD0765w	4	702203					-2
CNV4G	PFD0795w	4	727421					5
CNV4H	PFD0820w	4	749601					-3
CNV4I	PFD0835c	4	759267					7
CNV4J	PFD1150c	4	1093068					3
CNV4K	PFD1200c	4	1138422					-5
CNV4K	PFD1205w	4	1140613	*		*		-2
CNV4K	PFD1210w	4	1145464	*				0
CNV5A	PFE0040c	5	45544				Petersen1989	3
CNV5B	PFE0060w	5	64777					2
CNV5C	PFE0070w	5	77176					-2
CNV5D	PFE0290c	5	254890					-3
CNV5E	PFE0335w	5	286357					4
CNV5F	PFE0360c	5	303271		*			0
CNV5G	PFE0435c	5	364015					2
CNV5H	PFE0510c	5	441327					3
CNV5I	PFE0740c	5	616636					-2
CNV5J	PFE0825w	5	684782					-3
CNV5K	PFE0935c	5	777255					+/-2
CNV5L	PFE1035c	5	844079					2
CNV5M	PFE1060c	5	864754					-2
CNV5M	PFE1065w	5	869393		*			0
CNV5M	PFE1070c	5	872374		*			0
CNV5M	PFE1075c	5	876714		*			0

CNV5M	PFE1080w	5	878912	*	0
CNV5M	PFE1085w	5	883636	*	0
CNV5M	PFE1090w	5	886970	*	0
CNV5M	PFE1095w	5	891689	*	0
CNV5M	PFE1100w	5	896474	*	0
CNV5M	PFE1105c	5	898606	*	0
CNV5M	PFE1110w	5	901789	*	0
CNV5M	PFE1115c	5	903724	*	0
CNV5M	PFE1120w	5	922071	*	0
CNV5M	PFE1125w	5	937671	*	0
CNV5M	PFE1130w	5	940970	*	0
CNV5M	PFE1135w	5	943315	*	0
CNV5M	PFE1140c	5	944553	*	2
CNV5M	PFE1145w	5	951275	*	0
CNV5M	PFE1150w	5	960015	*	0
CNV5M	PFE1155c	5	964131	*	0
CNV5M	PFE1160w	5	967925	*	0
CNV5M	PFE1165c	5	970609	*	0
CNV5M	PFE1170w	5	974586	*	0
CNV5N	PFE1200w	5	1006168		0
CNV5N	PFE1210c	5	1011556		0
CNV5O	PFE1250w	5	1042752		0
CNV5O	PFE1255w	5	1048354		3
CNV5O	PFE1260c	5	1053187		4
CNV5P	PFE1280w	5	1068897		-3
CNV5Q	PFE1300w	5	1080389		-1
CNV5Q	PFE1325w	5	1107985		7
CNV5R	PFE1425c	5	1177121		1
CNV5S	PFE1440c	5	1184704		2
CNV5T	PFE1455w	5	1194146		+/3

CNV5U		PFE1490c	5	1221378			-1
CNV5V		PFE1505w	5	1227313			2
CNV5W		PFE1530c	5	1249158			+/-2
CNV6A	SD6L	PFF0050c	6	42658			0
CNV6A	SD6L	PFF0055w	6	44830			0
CNV6A	SD6L	PFF0060w	6	47859			-2
CNV6A	SD6L	PFF0065c	6	49295			0
CNV6B		PFF0090w	6	78484			-1
CNV6C		PFF0580w	6	506784			-2
CNV6D		PFF0790c	6	677227			2
CNV6E		PFF0870w	6	759800			-2
CNV6F		PFF0880c	6	765077			1
CNV6G		PFF0905w	6	779161			5
CNV6H		PFF0950w	6	826462			2
CNV6I		PFF1175c	6	991288			3
CNV6J		PFF1300w	6	1080159			-4
CNV6K		PFF1325c	6	1097976			1
CNV6K		PFF1330c	6	1100569			2
CNV6K		PFF1335c	6	1102294			4
CNV6L		PFF1350c	6	1116042			-4
CNV6M		PFF1377w	6	1177426	*		-2
CNV6O	SD6R	PFF1520w	6	1317300			0
CNV6O	SD6R	PFF1525c	6	1318725			0
CNV6O	SD6R	PFF1530c	6	1321710			1
CNV6O	SD6R	PFF1535w	6	1323868			0
CNV7A		MAL7P1.220	7	64957		Pologe1988	-6
CNV7A		MAL7P1.224	7	76518		Pologe1988	-2
CNV7A		MAL7P1.225	7	81315		Pologe1988	-2
CNV7A		MAL7P1.231	7	99203			-6
CNV7B	SD7L	MAL7P1.3	7	121670			0

CNV7B	SD7L	MAL7P1.4	7	123832		0
CNV7B	SD7L	MAL7P1.5	7	126831		-5
CNV7B	SD7L	PF07_0002	7	128277		0
CNV7C		MAL7P1.340	7	345999		+/-2
CNV7D		MAL7P1.30	7	508384		4
CNV7E		PF07_0042	7	522849		2
CNV7F		PF07_0070	7	792230		-2
CNV7G		MAL7P1.117	7	993963	*	1
CNV7G		PF07_0090	7	997521	*	0
CNV7G		MAL7P1.118	7	1000005	*	0
CNV7G		MAL7P1.119	7	1002864	*	0
CNV7G		MAL7P1.120	7	1006658	*	0
CNV7G		MAL7P1.122	7	1010940	*	0
CNV7G		PF07_0091	7	1012845	*	0
CNV7G		PF07_0092	7	1015166	*	0
CNV7H		MAL7P1.123	7	1022099		+/-2
CNV7I		MAL7P1.142	7	1136869		3
CNV7J		PF07_0112	7	1218647		-3
CNV7K		PF07_0116	7	1242663		2
CNV7L		MAL7P1.155	7	1277326		4
CNV7M		MAL7P1.163	7	1337430		-3
CNV8A		MAL8P1.160	8	84495		2
CNV8B		PF08_0136b	8	103514		-2
CNV8C		PF08_0122	8	275533		5
CNV8D		PF08_0119	8	292159		-4
CNV8E		PF08_0114	8	359555		2
CNV8F		PF08_0098	8	523719		4
CNV8G		PF08_0078	8	674408		3
CNV8H		MAL8P1.77	8	768652		2
CNV8I		MAL8P1.71	8	823019		4

CNV8J	PF08_0022	8	1144475						5
CNV8K	MAL8P1.22	8	1149992						2
CNV8L	MAL8P1.20	8	1167002						3
CNV8M	PF08_0008	8	1239465						5
CNV8N	MAL8P1.11	8	1249269						2
CNV8N	MAL8P1.10	8	1254144						3
CNV8O	PF08_0003	8	1298302						5
CNV8P	MAL8P1.1	8	1329237						-8
CNV8Q	MAL8P1.211	8	1367346						-2
CNV8R	MAL8P1.213	8	1370838						-5
CNV9A	PFI0060c	9	64322						-3
CNV9A	PFI0085c	9	82298						-3
CNV9B	PFI0360c	9	357167						2
CNV9C	PFI0415c	9	399956						3
CNV9D	PFI0525w	9	494826						-3
CNV9E	PFI0545w	9	510222						-2
CNV9F	PFI0565w	9	530723						3
CNV9G	PFI0785c	9	669481						3
CNV9H	PFI0895c	9	751880						-4
CNV9I	PFI0905w	9	762011						2
CNV9J	PFI0945w	9	793682						-4
CNV9K	PFI1030c	9	858198						2
CNV9L	PFI1080w	9	891652						-3
CNV9M	PFI1265w	9	1035937		*				-2
CNV9M	PFI1270w	9	1041105		*				0
CNV9N	PFI1380c	9	1137565						4
CNV9O	PFI1475w	9	1204383	*			*		2
CNV9P	PFI1520w	9	1261476						-1
CNV9P	PFI1525w	9	1263949						2
CNV9Q	PFI1710w	9	1378842	*			*	Spielmann	-2

CNV9Q	PFI1715w	9	1401341			*	Spielmann	-5
CNV9Q	PFI1720w	9	1406096			*	Spielmann	-4
CNV9Q	PFI1725w	9	1409593			*	Spielmann	-5
CNV9Q	PFI1730w	9	1416786			*	Spielmann	-6
CNV9Q	PFI1735c	9	1421634			*	Spielmann	-5
CNV9Q	PFI1740c	9	1427737			*	Spielmann	-5
CNV9Q	PFI1745c	9	1430866			*	Spielmann	0
CNV9Q	PFI1750c	9	1433709			*	Spielmann	-5
CNV9Q	PFI1755c	9	1437323			*	Spielmann	-5
CNV9Q	PFI1760w	9	1441211			*	Spielmann	-5
CNV9Q	PFI1765c	9	1444546			*	Spielmann	-4
CNV9Q	PFI1770w	9	1447426			*	Spielmann	0
CNV9Q	PFI1775w	9	1455059			*	Spielmann	-5
CNV9Q	PFI1780w	9	1459115		*	*	Spielmann	-5
CNV9Q	PFI1785w	9	1463018	*	*	*		-5
CNV9Q	PFI1790w	9	1466573		*	*		-1
CNV9Q	PFI1795c	9	1468970		*	*		-3
CNV9Q	PFI1800w	9	1472376	*	*	*		-5
CNV10A	PF10_0007	10	54657			*		0
CNV10A	PF10_0013	10	63593			*		0
CNV10A	PF10_0014	10	68099			*		0
CNV10A	PF10_0016	10	70684					-1
CNV10B	PF10_0023	10	99871					-1
CNV10C	PF10_0067	10	274513					2
CNV10D	PF10_0075	10	303523		*			0
CNV10D	PF10_0076	10	312640		*			0
CNV10D	PF10_0077	10	317465		*			0
CNV10D	PF10_0078	10	324581		*			0
CNV10D	PF10_0079	10	337027		*			0
CNV10D	PF10_0080	10	344316		*			0

CNV10E	PF10_0121	10	475944	-3
CNV10F	PF10_0134	10	541157	2
CNV10G	PF10_0136	10	547290	5
CNV10H	PF10_0190	10	801889	3
CNV10I	PF10_0217	10	941084	2
CNV10J	PF10_0252	10	1088314	5
CNV10K	PF10_0262	10	1116032	4
CNV10K	PF10_0263	10	1121734	-1
CNV10L	PF10_0268	10	1141594	4
CNV10M	PF10_0281	10	1181751	-5
CNV10M	PF10_0282	10	1183865	-7
CNV10M	PF10_0283	10	1185021	-8
CNV10M	PF10_0284	10	1192719	-6
CNV10M	PF10_0285	10	1196509	-7
CNV10M	PF10_0286	10	1198927	-8
CNV10M	PF10_0287	10	1201645	-6
CNV10M	PF10_0287	10	1201645	0
CNV10M	PF10_0288	10	1204552	-8
CNV10M	PF10_0290	10	1209379	0
CNV10M	PF10_0291	10	1212313	-8
CNV10M	PF10_0292	10	1219893	-8
CNV10M	PF10_0293	10	1223769	-3
CNV10M	PF10_0294	10	1226804	-8
CNV10M	PF10_0295	10	1231199	-8
CNV10M	PF10_0296	10	1235333	-8
CNV10N	PF10_0325	10	1340193	3
CNV10O	PF10_0327	10	1345522	2
CNV10P	PF10_0330	10	1355011	1
CNV10Q	PF10_0342	10	1392285	-1

CNV10Q		PF10_0343	10	1395715				*		-6
CNV10Q		PF10_0344	10	1401043				*		0
CNV10R		PF10_0348	10	1414244						-3
CNV10S		PF10_0355	10	1433639						-2
CNV10T		PF10_0357	10	1442734	*	*	*			3
CNV10U		PF10_0374	10	1533422					Scherf1992B	-3
CNV10U		PF10_0375	10	1549092					Scherf1992B	0
CNV10U		PF10_0376	10	1552334					Scherf1992B	0
CNV10U		PF10_0377	10	1554948					Scherf1992B	0
CNV10U		PF10_0378	10	1559255					Scherf1992B	0
CNV10U		PF10_0379	10	1563348					Scherf1992B	0
CNV10U		PF10_0380	10	1570261					Scherf1992B	0
CNV10U		PF10_0381	10	1574741					Scherf1992B	0
CNV10U		PF10_0382	10	1575709					Scherf1992B	2
CNV10U		PF10_0383	10	1582204					Scherf1992B	0
CNV10U		PF10_0384	10	1587263					Scherf1992B	-3
CNV10U		PF10_0385	10	1587836					Scherf1992B	0
CNV10U		PF10_0386	10	1588223					Scherf1992B	0
CNV10U		PF10_0387	10	1588505					Scherf1992B	0
CNV10U	SD10R	PF10_0388	10	1590403					Scherf1992B	0
CNV10U	SD10R	PF10_0390	10	1591972			*		Scherf1992B	-6
CNV10U	SD10R	PF10_0391	10	1594889					Scherf1992B	0
CNV10U	SD10R	PF10_0392	10	1597045					Scherf1992B	0
CNV11A		PF11_0012	11	54651			*	*		-4
CNV11A	SD11LA	PF11_0014	11	59939			*	*		-5
CNV11A	SD11LA	PF11_0015	11	61139						-4
CNV11A	SD11LB	PF11_0023	11	80865				*		0
CNV11A	SD11LB	PF11_0024	11	83024						0
CNV11A	SD11LB	PF11_0025	11	86024						0
CNV11A	SD11LB	PF11_0026	11	87465						0

CNV11A	PF11_0034	11	106296				3
CNV11B	PF11_0041	11	137187				3
CNV11C	PF11_0066	11	240150				-2
CNV11D	PF11_0072	11	259380				5
CNV11E	PF11_0092	11	346632				2
CNV11F	PF11_0108	11	407606	*			0
CNV11F	PF11_0109	11	410461	*			0
CNV11G	PF11_0113	11	426384				2
CNV11I	PF11_0124	11	456464				-2
CNV11J	PF11_0140	11	517647				2
CNV11K	PF11_0161	11	581973		*		0
CNV11L	PF11_0163	11	588784		*		0
CNV11M	PF11_0166	11	596745				5
CNV11N	PF11_0192	11	706468				4
CNV11O	PF11_0200	11	725296				5
CNV11P	PF11_0217	11	793070				2
CNV11Q	PF11_0224	11	815853				-4
CNV11R	PF11_0277	11	1041314				+/-1
CNV11R	PF11_0279	11	1051291	*			0
CNV11S	PF11_0281	11	1057124	*			0
CNV11T	PF11_0301	11	1122652				7
CNV11U	PF11_0317	11	1182886				-5
CNV11V	PF11_0341	11	1278990				6
CNV11W	PF11_0397	11	1523470				2
CNV11X	PF11_0416	11	1612838				8
CNV11Y	PF11_0447	11	1735868				7
CNV11Z	PF11_0457	11	1768833	*			0
CNV11Z	PF11_0458	11	1771423	*			0
CNV11AA	PF11_0472	11	1824952			*	0
CNV11BB	PF11_0476	11	1830938				2

CNV11CC	PF11_0478	11	1841148				7
CNV11CC	PF11_0479	11	1848627				8
CNV11CC	PF11_0480	11	1862840				8
CNV11DD	PF11_0488	11	1911352				5
CNV11EE	PF11_0514	11	2004880			*	+/-2
CNV12A	PFL0170w	12	180035				2
CNV12B	PFL0180w	12	185338				4
CNV12C	PFL0210c	12	205731				-3
CNV12D	PFL0465c	12	430682				3
CNV12E	PFL0555c	12	494634				3
CNV12F	PFL0615w	12	550393				5
CNV12G	PFL1130c	12	953999		*		0
CNV12G	PFL1135c	12	965052		*		0
CNV12H	PFL1155w	12	974949				-7
CNV12I	PFL1370w	12	1149777				4
CNV12J	PFL1395c	12	1174363				-3
CNV12K	PFL1465c	12	1252360				-3
CNV12L	PFL1605w	12	1377456				4
CNV12M	PFL1690w	12	1460647				-6
CNV12N	PFL1780w	12	1533189				-6
CNV12O	PFL2065c	12	1809238				-5
CNV12P	PFL2130w	12	1861740				-2
CNV12Q	PFL2255w	12	1947811				3
CNV12R	PFL2360w	12	2010645	*			0
CNV12S	PFL2405c	12	2049735			*	0
CNV12T	PFL2435w	12	2076140				2
CNV12U	PFL2455w	12	2089838				-5
CNV12V	PFL2545c	12	2162806				1
CNV12V	PFL2550w	12	2168987	*			0
CNV12V	PFL2555w	12	2171829	*			0

CNV12V	PFL2565w	12	2175691	*	*	*			0
CNV12V	PFL2570w	12	2181627	*	*	*			0
CNV12V	PFL2575c	12	2183799		*				-2
CNV12V	PFL2580w	12	2189168	*	*				0
CNV12V	PFL2590w	12	2196623				*		-6
CNV12V	PFL2595w	12	2199226						-3
CNV13A	MAL13P1.6	13	60738					Lavazec	0
CNV13A	MAL13P1.11	13	69051						-3
CNV13B	PF13_0073	13	83471						3
CNV13B	MAL13P1.59	13	86197						-2
CNV13C	MAL13P1.14	13	147888						6
CNV13D	MAL13P1.15	13	158663						3
CNV13E	PF13_0022	13	242512						-2
CNV13F	PF13_0036	13	332245						3
CNV13G	PF13_0063	13	490970						-2
CNV13H	MAL13P1.66	13	560838						2
CNV13G	PF13_0082	13	623405						+/-4
CNV13H	MAL13P1.84	13	669755						+/-3
CNV13I	MAL13P1.87	13	699272						4
CNV13J	MAL13P1.97	13	767304						-2
CNV13K	MAL13P1.107	13	824509						-5
CNV13L	PF13_0138	13	1022224						3
CNV13M	PF13_0146	13	1073648						2
CNV13N	MAL13P1.162	13	1277766						-2
CNV13O	PF13_0173	13	1334144						4
CNV13O	PF13_0174	13	1336845						+/-2
CNV13P	MAL13P1.176	13	1433842						5
CNV13P	PF13_0198	13	1445780						5
CNV13Q	MAL13P1.217	13	1721027						+/-2
CNV13R	MAL13P1.261	13	2064372						5

CNV13S	PF13_0275	13	2121104			-5
CNV13T	PF13_0288	13	2175584			7
CNV13U	PF13_0304	13	2262816			-4
CNV13V	MAL13P1.297	13	2390181			2
CNV13W	PF13_0347	13	2631686			-2
CNV13X	PF13_0358	13	2722142			4
CNV13Y	PF13_0361	13	2771872			2
CNV13Z	MAL13P1.480	13	2818215		Wellems1987	-3
CNV14A	PF14_0009	14	30547	*		-6
CNV14B	PF14_0016	14	53574			-5
CNV14C	PF14_0034	14	134577			3
CNV14D	PF14_0037	14	144323			-2
CNV14E	PF14_0082	14	320568			3
CNV14F	PF14_0087	14	354202			-2
CNV14G	PF14_0101	14	410357			6
CNV14H	PF14_0105	14	430276			2
CNV14I	PF14_0134	14	539353			2
CNV14J	PF14_0144	14	591406			-2
CNV14K	PF14_0230	14	974640			-3
CNV14L	PF14_0260	14	1103115			-1
CNV14M	PF14_0265	14	1127979			-3
CNV14N	PF14_0268	14	1136965			-2
CNV14O	PF14_0274	14	1165391			-1
CNV14P	PF14_0277	14	1173003			-4
CNV14Q	PF14_0336	14	1436605			1
CNV14R	PF14_0374	14	1610257			-3
CNV14S	PF14_0473	14	2039774			-2
CNV14T	PF14_0482	14	2079356			2
CNV14U	PF14_0490	14	2113719			-2
CNV14V	PF14_0558	14	2402899			5

CNV14W	PF14_0563	14	2421813			-1
CNV14X	PF14_0576	14	2462149			3
CNV14Y	PF14_0653	14	2813652			5
CNV14Z	PF14_0656	14	2829129			5
CNV14AA	PF14_0666	14	2865571	*		0
CNV14AA	PF14_0667	14	2867207	*		0
CNV14BB	PF14_0669	14	2878324			-1
CNV14CC	PF14_0732	14	3139996	*	*	0
CNV14CC	PF14_0733	14	3142633	*	*	0
CNV14CC	PF14_0734	14	3143851	*	*	0
CNV14CC	PF14_0735	14	3147972			5
CNV14DD	PF14_0747	14	3196317	*	*	-1
CNV14EE	PF14_0764	14	3263807			-3

[a] Named according to chromosome number in ascending letter from left to right for CNVs and left (L) or right (R) arm for SDs. Significant genes that were adjacent to other significant genes in this study or previously published CNVs were assigned the same CNV name.

[b] According to PlasmoDB Version 5.5.

[c] Marked as a * if they were recorded in the CGH studies indicated by the column heading. Kidgell, [1]; Ribacke, [2]; Bozdech, [3]; Carret, [4].

[d] Marked by the study name where they were recorded. Pologe1988, [5]; Pologe1990, [6]; Scherf1992A, [7]; Scherf1992B, [8], Petersen1989, [9]; Lavazec, [10]; Wellems, [11]; Spielmann, [12]

[e] Defined as a CNV from the data in this study if significant variation between field strains in CGH data (P<0.001, >1.5-fold difference from 3D7 in at least 2 field strains). +/- n indicates the number of strains (n) different from 3D7 and the direction of these differences.

Note: Since this table was constructed, three more studies on CNVs in laboratory isolates have been published [13–15]. Their results are not included here.

References for Table S2

1. Kidgell C, Volkman SK, Daily JP, Borevitz JO, Plouffe D et al. (2006) A systematic map of genetic variation in *Plasmodium falciparum*. PLoS Pathog 2: e57.

2. Ribacke U, Mok BW, Wirta V, Normark J, Lundeberg J et al. (2007) Genome wide gene amplifications and deletions in *Plasmodium falciparum*. Mol Biochem Parasitol 155: 33-44.

3. Bozdech Z, Llinas M, Pulliam BL, Wong ED, Zhu JC et al. (2003) The transcriptome of the intraerythrocytic developmental cycle of *Plasmodium falciparum*. PLoS Biol 1: 85-100.

4. Carret CK, Horrocks P, Konfortov B, Winzeler EA, Qureshi M et al. (2005) Microarray-based comparative genomic analyses of the human malaria parasite *Plasmodium falciparum* using Affymetrix arrays. Mol Biochem Parasitol 144: 177-186.

5. Pologe LG, Ravetch JV (1988) Large deletions result from breakage and healing of *P. falciparum* chromosomes. Cell 55: 869-874.

6. Pologe LG, de Bruin D, Ravetch JV (1990) A and T homopolymeric stretches mediate a DNA inversion in *Plasmodium falciparum* which results in loss of gene expression. Mol Cell Biol 10: 3243-3246.

7. Scherf A, Mattei D (1992) Cloning and characterization of chromosome breakpoints of *Plasmodium falciparum*: breakage and new telomere formation occurs frequently and randomly in subtelomeric genes. Nucleic Acids Res 20: 1491-1496.

8. Scherf A, Carter R, Petersen C, Alano P, Nelson R et al. (1992) Gene inactivation of Pf11-1 of *Plasmodium falciparum* by chromosome breakage and healing: identification of a gametocyte-specific protein with a potential role in gametogenesis. EMBO J 11: 2293-2301.

9. Petersen C, Nelson R, Magowan C, Wollish W, Jensen J et al. (1989) The mature erythrocyte surface antigen of *Plasmodium falciparum* is not required for knobs or cytoadherence. Mol Biochem Parasitol 36: 61-65.

10. Lavazec C, Sanyal S, Templeton TJ (2007) Expression switching in the stevor and Pfmc-2TM superfamilies in *Plasmodium falciparum*. Mol Microbiol 64: 1621-1634.

11. Wellems TE, Walliker D, Smith CL, Rosario VE, Maloy WL et al. (1987) A histidine-rich protein gene marks a linkage group favored strongly in a genetic cross of *Plasmodium falciparum*. Cell 49: 633-642.

12. Spielmann T, Hawthorne PL, Dixon MW, Hannemann M, Klotz K et al. (2006) A cluster of ring stage-specific genes linked to a locus implicated in cytoadherence in *Plasmodium falciparum* codes for PEXEL-negative and PEXEL-positive proteins exported into the host cell. Mol Biol Cell 17: 3613-3624.

13. Jiang H, Yi M, Mu J, Zhang L, Ivens A et al. (2008) Detection of genome-wide polymorphisms in the AT-rich *Plasmodium falciparum* genome using a high-density microarray. BMC Genomics 9: 398.

14. Cheeseman IH, Gomez-Escobar N, Carret CK, Ivens A, Stewart LB et al. (2009) Gene copy number variation throughout the *Plasmodium falciparum* genome. BMC Genomics 10: 353.

15. Dharia NV, Sidhu AB, Cassera MB, Westenberger SJ, Bopp SE et al. (2009) Use of high-density tiling microarrays to identify mutations globally and elucidate mechanisms of drug resistance in *Plasmodium falciparum*. Genome Biol 10: R21.
