# Supplementary material for: Comparative Transcriptional and Genomic Analysis of Plasmodium falciparum Field Isolates
Source: PLoS Pathog. 2009 Oct 30;5(10):e1000644. doi: 10.1371/journal.ppat.1000644 (PMC2764095; doi:10.1371/journal.ppat.1000644)
Supplement: Table S3 — Differential expression results from this study for genes associated with pregnancy malaria in previous studies. (0.07 MB DOC) [file ppat.1000644.s013.doc]

**Table S3**. Differential expression results from this study for genes associated with pregnancy malaria in previous studies.

| **Gene ID** |  | **Previous studies** | | |  | **This study** | |
| --- | --- | --- | --- | --- | --- | --- | --- |
|  |  | **Comparisons^a^** | **Result** | **References*^b^*** |  | **Result^c^** | **Distance from CNV (kb)** |
| PFA0700c |  | Pvs3D7,PPPE | Higher | Nd,Fd |  | Field>Lab | CNV1F |
| PFB0105c |  | Pvs3d7 | Higher | Nd |  | Lab>Field | CNV2A |
| PFB0115w |  | Pvs3D7,MPvsCP,  PvsCP,PPPA | Higher | Fr,Nd |  | Lab>Field | 9.2 |
| PFB0870w |  | PPPE | Higher | Fd |  | n.s. | 5.9 |
| PFB0888w |  | PPPA | Higher | Fd |  | n.s. | 23 |
| PFC0110w |  | Pvs3D7 | Higher | Nd |  | Field>Lab | CNV3B |
| PFC0715c |  | PPPE | Higher | Fd |  | n.s. | 19 |
| PFC0850c |  | PPPE | Higher | Fd |  | n.s. | 11 |
| PFD0690c |  | PPPE | Higher | Fd |  | n.s. | 39 |
| PFD1120c |  | Pvs3D7,PvsCP | Lower | Nd |  | Field>Lab,  Var in Field | 17 |
| PFD1140w |  | Pvs3D7,PvsCP,PvsCP | Higher | Fr,Nd |  | Lab>Field | 7 |
| PFF0435w |  | MPvsCP | Higher | Fr |  | n.s. | 143 |
| MAL7P1.225 |  | PvsCP | Higher | Fr |  | Lab>Field | CNV7A |
| PFI1785w |  | Pvs3D7,MPvsCP,  PvsCP,PPPE | Higher | Fr,Nd,Fd |  | n.s. | CNV9Q |
| PF10_0013 |  | PvsCP | Higher | Fr |  | n.s. | CNV10A |
| PF10_0232 |  | PPPA | Higher | Fd |  | Lab>Field | 61 |
| PF10_0344 |  | Pvs3d7 | Higher | Nd |  | n.s. | CNV10Q |
| PF10_0350 |  | Pvs3D7,PvsCP | Lower | Nd |  | Field>Lab | 7.4 |
| PF10_0351 |  | Pvs3D7 | Higher | Nd |  | n.s. | 8.8 |
| PF11_0437 |  | PPPA | Higher | Fd |  | n.s. | 30 |
| PFL0260c |  | Pvs3d7 | Lower | Nd |  | n.s. | 21 |
| PFL1385c |  | MPvsCP | Higher | Fr |  | n.s. | 12 |
| PFL2505c |  | PPPA | Higher | Fd |  | n.s. | 29 |
| PF13_0162 |  | PPPA | Higher | Fd |  | n.s. | 23 |
| MAL13P1.320 |  | MPvsCP,PvsCP | Higher | Fr |  | n.s. | 78 |
| MAL13P1.470 |  | MPvsCP | Higher | Fr |  | n.s. | 7.6 |
| PF14_0010 |  | Pvs3d7 | Higher | Nd |  | Field>Lab | 4.7 |
| PF14_0016 |  | PPPA | Higher | Fd |  | n.s. | CNV14B |
| PF14_0260 |  | PPPA | Higher | Fd |  | n.s. | CNV14L |
| PF14_0507 |  | PPPA | Higher | Fd |  | n.s. | 77 |
| PF14_0616 |  | PPPE | Higher | Fd |  | Lab>Field  Var in Field | 169 |

1. Comparisons between experimental groups. Pvs3D7, mRNA expression levels in placental parasites vs. 3D7 cultured parasites; PvsCP, mRNA expression levels in placental parasites vs. parasites from the peripheral circulation of children; MPvsCP, mRNA expression levels in parasites from the periphery of pregnant women vs. parasites from the peripheral circulation of children; PPPE, protein expression found exclusively in placental parasites cf. parasites from the peripheral circulation of children; PPPA, PPPE, protein expression found to be more abundant in placental parasites cf. parasites from the peripheral circulation of children.
2. Fd, Fried et al. (2007); Fr, Francis et al. (2008); Nd, Ndam et al. 2008
3. Field > Lab, significantly higher in field isolates vs. laboratory isolates; Lab<Field, the reverse of this; Var in Field, significantly variable among field isolates, n.s.; not significantly for any of the above.
